# Deadlock-Resistant Safety-Critical Mobile Robot Navigation Using Guiding Vector Fields and Model Predictive Control

**DOI:** 10.3390/s26196304

**Published:** 2026-10-06

**Authors:** Liang Gao, Yongfeng Gao, Xinhui Zhao, Xuefeng Li, Mingde He, Yuhu Wu

**Affiliations:** 1 Key Laboratory of Intelligent Control and Optimization for Industrial Equipment of the Ministry of Education, School of Control Science and Engineering, Dalian University of Technology, Dalian 116024, China; gliang@mail.dlut.edu.cn (L.G.); gaoyongfeng@dlut.edu.cn (Y.G.); 2732153007@mail.dlut.edu.cn (X.Z.); hemingde@mail.dlut.edu.cn (M.H.); wuyuhu@dlut.edu.cn (Y.W.); 2 Department of Computing Science and Artificial Intelligence, Liaoning Normal University, No. 1 Liushu South Street, Ganjingzi District, Dalian 116081, China

**Keywords:** mobile robot navigation, deadlock avoidance, guiding vector field, model predictive control, discrete-time control barrier function

## Abstract

This paper investigates the safe navigation of mobile robots in complex-static-obstacle environments and proposes a deadlock-resistant navigation control method. First, convex polygonal obstacles are modeled using the Log-Sum-Exp (LSE) function. Goal and obstacle avoidance guiding vector fields (GVFs) are then constructed, and an obstacle-bypassing method is developed using the goal direction and local obstacle geometry. The goal and obstacle avoidance GVFs are further blended to construct a navigation GVF, for which a regional sufficient condition excluding complete cancellation is established. On this basis, a safe robot navigation controller is designed by combining the navigation GVF with a model predictive control (MPC) framework incorporating discrete-time control barrier function (DCBF) constraints. We also give conditions for obstacle avoidance throughout each sampling interval under bounded one-step position prediction errors. Numerical simulations show that, in the tested scenarios, wherethe tested artificial potential field (APF) and conventional MPC-DCBF configurations fail to complete the navigation task, the proposed methodcan bypass obstacles and reach the goal. Hardware experiments further validate the effectiveness of the proposed method on a physical robot platform in a preconfigured environment.

## 1. Introduction

Autonomous mobile robots have been widely deployed in intelligent manufacturing [1], warehouse logistics [2], inspection [3], and service robotics [4]. Reliable autonomous navigation requires the continuous generation of motion commands from real-time robot-state and environment information, together with closed-loop control that drives the robot to a prescribed goal region while satisfying kinematic, control input, and collision safety constraints. In complex-obstacle environments, when progress toward the goal is obstructed by an obstacle, goal-seeking and collision avoidance may impose conflicting requirements, causing the robot to remain collision-free while becoming stationary outside the goal region [5]. We refer to this phenomenon as navigation deadlock. Therefore, avoiding navigation deadlock while preserving collision safety and goal-reaching behavior is an important problem in the point-to-point navigation of mobile robots.

Artificial potential field (APF) methods are widely used for mobile robot navigation and obstacle avoidance because of their simple computation and suitability for online implementation [6,7,8]. A typical APF method generates the robot motion direction by superimposing a goal attraction term and obstacle repulsion terms. When the attractive and repulsive effects balance, however, the resultant navigation direction may approach zero and create a local minimum [9,10], preventing further progress toward the goal [11,12]. Thus, superposed attraction and repulsion alone do not provide persistent tangential motion for bypassing a blocking obstacle.

Compared with local potential field navigation, model predictive control (MPC) can explicitly account for robot kinematics, control input constraints, and state constraints over a finite prediction horizon and is therefore widely used for constrained mobile robot navigation [13,14,15]. Control barrier functions (CBFs) characterize safe sets through safety inequalities and can be embedded in online optimization controllers to impose collision safety constraints [16,17,18]. Such constraints, however, primarily exclude unsafe motion and do not specify how the robot should pass a blocking obstacle. Consequently, goal-directed control subject only to collision safety constraints may admit undesirable equilibria [19,20,21], and safety-constrained MPC can encounter deadlock in particular navigation scenarios [5,22]. These observations motivate a navigation reference that supplies persistent obstacle-bypassing motion while remaining compatible with predictive safety constraints.

To address navigation deadlock, the robot needs an appropriate tangential obstacle avoidance motion when direct progress toward the goal is blocked. Guiding vector field (GVF) methods typically construct convergence and tangential components from the local geometry of a reference curve, allowing a robot to converge toward the curve while retaining a motion component along its tangent [23,24,25,26]. This property provides a useful basis for designing obstacle avoidance GVFs for deadlock-resistant navigation. By constructing an obstacle avoidance GVF with both convergence and tangential components, tangential bypass motion can be maintained while the robot satisfies the obstacle avoidance requirement, thereby reducing the risk of navigation deadlock. Existing GVF methods, however, are developed primarily for tracking prescribed paths or curves. Their use in point-to-point robot navigation additionally requires determining the tangential motion direction during obstacle avoidance.

For a given obstacle, two candidate tangential motions generally exist, corresponding to clockwise and counterclockwise traversal. These are referred to here as the two bypass directions (also called circulation directions). If the circulation direction is treated as a prescribed design choice, the selected direction may be poorly aligned with subsequent goal approach. Conversely, if the bypass direction is repeatedly switched according to instantaneous geometric information during an avoidance maneuver, the tangential reference direction reverses with it. Therefore, a key issue in applying GVFs to point-to-point navigation is to determine an appropriate clockwise or counterclockwise bypass direction for each obstacle and retain the selected direction until the corresponding obstacle has been cleared. As the robot moves past the obstacle, the influence of the associated obstacle avoidance GVF should also be reduced so that navigation gradually returns to the goal GVF.

These issues become more important in multi-obstacle environments. The smooth interpolation of local vector fields over a cell decomposition has been used to construct collision-free feedback laws [27]. In the setting considered here, a goal GVF and obstacle avoidance GVFs associated with individual obstacles are smoothly combined into a unified navigation GVF. However, even when each local GVF is nonzero, the weighted combination can be weakened or vanish if their directions conflict. A vanishing field eliminates the navigation reference and can thereby contribute to navigation deadlock. This possibility motivates both a blending design and an analysis of the conditions under which complete directional cancellation can be excluded in prescribed regions.

Motivated by the above observations, this paper proposes a deadlock-resistant safe navigation strategy for mobile robots. First, convex polygonal obstacles are modeled using the Log-Sum-Exp (LSE) function, and the resulting continuous geometric information is used to construct the goal and obstacle avoidance GVFs. The bypass direction is selected online according to the geometric relationship between the goal-seeking direction and the two candidate local bypass directions around the obstacle. When this geometric relationship does not clearly distinguish between the two candidates, the previously selected bypass direction is retained. The selected direction is then kept fixed throughout the entire bypass maneuver to avoid repeated direction switching. The goal GVF and the obstacle avoidance GVFs are further blended smoothly to construct a multi-obstacle navigation GVF, and a regional condition under which the blended field remains nonzero is analyzed. On this basis, the reference velocity generated by the navigation GVF is used as the tracking target of MPC, while discrete-time control barrier function (DCBF) constraints are imposed to enforce collision safety. Finally, numerical simulations and hardware experiments are conducted to validate the proposed method.

The main contributions are summarized as follows:

1. A deadlock-resistant obstacle-bypassing method is proposed to mitigate navigation deadlock caused by conflicts between goal-seeking and obstacle avoidance. Unlike methods with a predefined bypass direction [20,21], the proposed method selects the bypass direction online based on the current local geometry and retains the previous direction when the selection criterion is ambiguous, thereby avoiding repeated direction-switching and reducing the risk of navigation deadlock.
2. A GVF design method for robot navigation is proposed to address possible cancellation when multiple local obstacle avoidance GVFs participate simultaneously. For a prescribed region, a common positive projection condition on the participating GVFs is established as a sufficient condition for the blended navigation GVF to remain nonzero.
3. A GVF-based MPC-DCBF navigation method is proposed. A sufficient condition for collision safety is established by accounting for one-step position prediction errors and inter-sample robot motion, together with an LSE-based compatibility condition between the curvature of the bypass curve and the robot’s turning capability.

## 2. Preliminaries and Problem Formulation

This section first presents the sampled-data model of the robot and reviews the basic definition of DCBFs. It then describes the convex polygonal obstacles and their associated safety constraints and defines the navigation objective. Finally, the deadlock-resistant safe navigation problem investigated in this paper is formulated.

### 2.1. Sampled-Data Robot Model

Consider a mobile robot moving in a bounded two-dimensional workspace $W\subset R^{2}$. Let $p\left(t\right)={\left[p_{x}\left(t\right),p_{y}\left(t\right)\right]}^{T}$ denote its position, θ(t)∈(−π,π] its heading angle, and v(t) and ω(t) its linear and angular velocities, respectively. The admissible state set is denoted by $X\subseteq W\times S^{1}$. Let $v_{\min},v_{\max}$ and $\omega_{\min},\omega_{\max}$ be the prescribed lower and upper bounds on these velocities, with $\omega_{\min}<0<\omega_{\max}$. Define

$$u_{\min}={\left[v_{\min},\omega_{\min}\right]}^{T},\;u_{\max}={\left[v_{\max},\omega_{\max}\right]}^{T},$$

and the admissible input set

$$U=\{z\in R^{2}:u_{\min}\leq z\leq u_{\max}\}.$$

The state and control input are defined, respectively, as

(1) $$x\left(t\right)={\left[\begin{array}{ccc} p_{x}\left(t\right) & p_{y}\left(t\right) & \theta (t) \end{array}\right]}^{T}\in X,\;u\left(t\right)={\left[\begin{array}{cc} v(t) & \omega (t) \end{array}\right]}^{T}\in U.$$

The continuous-time nonholonomic kinematics of the robot are given by

(2) $$\dot{x}\left(t\right)=G\left(\theta \left(t\right)\right)u\left(t\right),\;G\left(\theta \right):=\left[\begin{array}{cc} \cos\theta & 0\\ \sin\theta & 0\\ 0 & 1 \end{array}\right].$$

The corresponding planar velocity is

(3) $$\nu \left(x,u\right)=v{\left[\begin{array}{cc} \cos\theta & \sin\theta \end{array}\right]}^{T}.$$

Since the robot has no lateral velocity input, its translational motion is restricted to the current heading direction.

The robot evolves continuously, whereas state measurements are taken with a sampling period $T_{s}>0$ at the instants

(4) $$t_{k}=kT_{s},\;k\in Z_{\geq 0}.$$

Write $x_{k}:=x\left(t_{k}\right)$, $p_{k}:=p\left(t_{k}\right)$, and $\theta_{k}:=\theta \left(t_{k}\right)$. Let $u_{k}$ denote the command applied over the *k*-th sampling interval.

The control input is implemented through a zero-order hold between consecutive sampling instants

(5) $$u\left(t\right)=u_{k},\;t\in \left[t_{k},t_{k+1}\right).$$

Control updates may occur at a subset of the sampling instants, with the previously applied input retained between updates. For online prediction, the continuous-time model in (2) is discretized using the forward Euler method with sampling period $T_{s}$, yielding

(6) $$x_{k+1}=f_{d}\left(x_{k},u_{k}\right)=x_{k}+T_{s}G\left(\theta_{k}\right)u_{k}.$$

At sampling instant $t_{k}$, $u_{k-1}$ denotes the input applied over the preceding sampling interval. At k=0, $u_{-1}$ is the prescribed prior input, whereas $u_{0}$ is the command applied over $\left[t_{0},t_{1}\right)$. Define the input increment as $\Delta u_{k}=u_{k}-u_{k-1}$. Let $\Delta v_{\max}$ and $\Delta \omega_{\max}$ denote the nonnegative bounds on the magnitudes of the corresponding input increments, and define the symmetric limits

$$\Delta u_{\min}=-{\left[\Delta v_{\max},\Delta \omega_{\max}\right]}^{T},\;\Delta u_{\max}={\left[\Delta v_{\max},\Delta \omega_{\max}\right]}^{T}.$$

The control input and its increment satisfy

(7) $$u_{\min}\leq u_{k}\leq u_{\max},\;\Delta u_{\min}\leq \Delta u_{k}\leq \Delta u_{\max}.$$

### 2.2. DCBF Preliminaries

To formulate the collision safety constraints used below, consider the discrete-time system (6) and a continuously differentiable function h:X→R. Its zero-superlevel set is defined as

(8) S={x∈X:h(x)≥0}.

**Definition** **1** (DCBF [17])**.** *If there exists a constant γ∈(0,1] such that, for every $x_{k}\in S$, there exists $u_{k}\in U$ satisfying*

(9) $$h\;\left(f_{d}\left(x_{k},u_{k}\right)\right)\geq \left(1-\gamma \right)h\left(x_{k}\right),$$

*then h can be used to construct a DCBF safety constraint for (6).*

The corresponding set of DCBF-admissible inputs is

(10) $$U_{h}\left(x_{k}\right)=\left\{u_{k}\in U:h\;\left(f_{d}\left(x_{k},u_{k}\right)\right)\geq \left(1-\gamma \right)h\left(x_{k}\right)\right\}.$$

If $x_{0}\in S$ and $u_{k}\in U_{h}\left(x_{k}\right)$ along the closed-loop trajectory, the recursive application of (9) yields

(11) $$x_{k}\in S,\;\forall k\in Z_{\geq 0}.$$

Thus, the DCBF condition guarantees the forward invariance of the safe set at the sampling instants.

### 2.3. Obstacle Modeling and Navigation Constraints

Consider *M* static convex polygonal obstacles in the workspace W, and define the obstacle index set M={1,…,M}. Let $O_{i}\subset R^{2}$ denote the *i*-th obstacle. The union of all obstacles is denoted by

(12) $$O=\underset{i\in M}{⋃}O_{i}.$$

**Assumption** **1.** *Each $O_{i}$ is a bounded convex polygon with a nonempty interior. A nonconvex obstacle is represented through convex decomposition as a finite union of convex polygons, and each convex component is treated as an individual convex polygonal obstacle.*

Suppose that obstacle $O_{i}$ has $N_{i}$ edges, and define its edge index set as $N_{i}=\left\{1,\ldots ,N_{i}\right\},\;\;i\in M$. For edge $l\in N_{i}$, let $a_{il}\in R^{2}$ be its unit outward normal and $b_{il}\in R$ the scalar offset locating its supporting line. The associated signed half-space function is $\ell_{il}\left(p\right)=a_{il}^{T}p+b_{il}$. Then, $O_{i}$ admits the half-space representation

(13) $$O_{i}=\left\{p\in R^{2}:\begin{array}{c} \ell_{il}\left(p\right)=a_{il}^{T}p+b_{il}\leq 0,\;\;l\in N_{i} \end{array}\right\}.$$

The unit normals satisfy

(14) $$∥a_{il}∥=1,\;i\in M,\;l\in N_{i}.$$

Model the robot as a disk centered at p with radius $r_{R}>0$, and let $d_{s}\geq 0$ denote the prescribed safety buffer between the robot and the obstacles. For the disk model, define

(15) $$\delta =r_{R}+d_{s}.$$

The quantity δ is the equivalent center-to-obstacle distance threshold.

For nonempty planar sets *A* and *B*, define their Euclidean distance by $\mathrm{dist}\left(A,B\right)=\inf_{a\in A,\;b\in B}∥a-b∥$. For a point p, write dist(p,B)=dist({p},B). The minimum distance between the robot boundary and obstacle $O_{i}$ is $d_{\mathrm{edge},i}\left(p\right)=\mathrm{dist}\left(p,O_{i}\right)-r_{R}$ whenever the two sets are disjoint. Hence, the physical edge-to-edge safety requirement is equivalently written as

(16) $$d_{\mathrm{edge},i}\left(p\right)\geq d_{s}\;⟺\;\mathrm{dist}\left(p,O_{i}\right)\geq \delta ,\;i\in M.$$

The corresponding safe set is defined as

(17) $$S_{\delta }=\left\{x\in X:\mathrm{dist}(p,O_{i})\geq \delta ,\;\forall i\in M\right\}.$$

Given a goal position $p_{g}\in W$, define the distance between the robot and the goal as

(18) $$r_{g}\left(p\right)=∥p-p_{g}∥.$$

For a given position tolerance $\varepsilon_{g}>0$, define the goal set as

(19) $$G_{\varepsilon_{g}}=\left\{x\in S_{\delta }:r_{g}\left(p\right)\leq \varepsilon_{g}\right\}.$$

Because this paper considers point-to-point navigation, only the terminal position is prescribed, and no terminal heading constraint is imposed. It is further assumed that $x_{0}\in S_{\delta }$ and that the goal set is nonempty, i.e., $G_{\varepsilon_{g}}\neq ⌀$.

### 2.4. Problem Statement

Accordingly, the navigation design must reduce $r_{g}\left(p\right)$ when goal-directed motion is unobstructed and provide a consistent bypass motion when an obstacle blocks direct progress. For each obstacle requiring a bypass maneuver, the bypass direction is selected according to the relative geometry among the robot, goal, and obstacle when the local geometry clearly distinguishes between the clockwise and counterclockwise candidates. Otherwise, the previously selected bypass direction is retained; if none exists, the preset initial direction is used. The selected direction is kept fixed throughout the corresponding bypass maneuver.

Safety constraints are primarily used to ensure obstacle avoidance and do not directly specify a circulation direction. Let Φ denote the navigation GVF to be designed: it assigns a nominal planar velocity to each position in a chosen admissible position domain contained in W. Denote its velocity output by $\nu^{\mathrm{GVF}}$, so that

(20) $$\nu^{\mathrm{GVF}}\left(p\right)=\Phi \left(p\right).$$

This nominal velocity specifies the desired navigation motion and is used to construct the controller’s tracking reference below.

In a multi-obstacle environment, the circulation directions associated with different obstacles may conflict, reducing the blended nominal GVF velocity or even causing it to vanish:

(21) $$\Phi \left(p\right)=0,\;r_{g}\left(p\right)>\varepsilon_{g}.$$

In this case, the robot has not entered the goal set, but the navigation GVF can no longer provide an effective navigation direction, which may lead to navigation deadlock. It is therefore necessary to characterize the conditions under which the nominal GVF velocity remains nonzero in prescribed regions outside the goal set.

Moreover, (3) shows that the robot cannot directly realize every planar GVF velocity Φ(p). Its inputs *v* and ω must adjust the speed and heading to follow a reference derived from Φ(p) while satisfying (7). Thus, a sampled-data controller must track this reference subject to motion and collision safety constraints. These considerations lead to the following problem.

**Problem** **1** (Deadlock-resistant safe navigation)**.** *Consider the mobile robot described by (6) and the static polygonal obstacle environment described by (13). For a given initial state $x_{0}\in S_{\delta }$, design a GVF ***Φ*** on an admissible navigation domain within W and a sampled-data feedback law*

(22) $$u_{k}=\pi_{\mathrm{MPC}}\left(x_{k},u_{k-1}\right)$$

*such that the closed-loop robot achieves deadlock-resistant safe navigation toward the goal set $G_{\varepsilon_{g}}$ while satisfying the constraint (7) and (16).*

## 3. GVF Construction

This section models convex polygonal obstacles using LSE functions and relates the model to the physical safety distance and reference-curve curvature. It then constructs the navigation GVF and analyzes its regional nonvanishing.

### 3.1. LSE Modeling of Convex Polygonal Obstacles

For the *i*-th convex polygonal obstacle $O_{i}$ described by (13), where i∈M, choose an LSE smoothing parameter $\kappa_{i}>0$ and use the signed half-space functions $\ell_{il}$ to define

(23) $$F_{i}\left(p\right)=\frac{1}{\kappa_{i}}\ln\left(\frac{1}{N_{i}}\sum_{l\in N_{i}}\exp\left(\kappa_{i}\ell_{il}\left(p\right)\right)\right),\;\kappa_{i}>0.$$

Define the normalized exponential weights

(24) $$\begin{array}{c} \lambda_{il}\left(p\right)=\frac{\exp\left(\kappa_{i}\ell_{il}\left(p\right)\right)}{\sum_{q\in N_{i}}\exp\left(\kappa_{i}\ell_{iq}\left(p\right)\right)}. \end{array}$$

With these weights, the gradient of $F_{i}$ is

(25) $$\begin{array}{c} \nabla F_{i}\left(p\right)=\sum_{l\in N_{i}}\lambda_{il}\left(p\right)a_{il}. \end{array}$$

Since $\lambda_{il}\left(p\right)\geq 0$, $\sum_{l\in N_{i}}\lambda_{il}\left(p\right)=1$, $\nabla F_{i}\left(p\right)$ is a convex combination of the unit outward normals of the polygon edges. Therefore, $F_{i}$ defines an LSE model of the polygonal obstacle, and its gradient supplies continuous geometric information for constructing obstacle normals and circulation directions.

To quantify the effect of $\kappa_{i}$, define

$$m_{i}\left(p\right)=\max_{l\in N_{i}}\ell_{il}\left(p\right),\;\eta_{i}=\frac{\ln N_{i}}{\kappa_{i}}.$$

The standard LSE bounds give, for every $p\in R^{2}$,

(26) $$\max_{l\in N_{i}}\ell_{il}\left(p\right)-\frac{\ln N_{i}}{\kappa_{i}}\leq F_{i}\left(p\right)\leq \max_{l\in N_{i}}\ell_{il}\left(p\right).$$

Consequently,

(27) $$O_{i}\subseteq \left\{p:F_{i}\left(p\right)\leq 0\right\}\subseteq \left\{p:\max_{l\in N_{i}}\ell_{il}\left(p\right)\leq \frac{\ln N_{i}}{\kappa_{i}}\right\}.$$

Equation (27) shows that $F_{i}\left(p\right)=0$ defines a smooth conservative enclosure of the original convex polygon. The quantity $\eta_{i}$ bounds the function-value error $0\leq m_{i}\left(p\right)-F_{i}\left(p\right)\leq \eta_{i}$.

Let $\nabla^{2}F_{i}$ denote the Hessian of $F_{i}$ with respect to p. Differentiating (25) yields

(28) $$\nabla^{2}F_{i}\left(p\right)=\kappa_{i}\sum_{l\in N_{i}}\lambda_{il}\left(p\right)\left(a_{il}-\nabla F_{i}\left(p\right)\right){\left(a_{il}-\nabla F_{i}\left(p\right)\right)}^{T}.$$

For symmetric matrices, write A⪯B when B−A is positive semidefinite, and let $I_{2}$ denote the two-dimensional identity matrix. Since (28) is a weighted covariance matrix,

(29) $$0⪯\nabla^{2}F_{i}\left(p\right)⪯\kappa_{i}I_{2},\;∥\nabla F_{i}\left(p\right)∥\leq 1.$$

It follows from (29) that $F_{i}$ is convex and globally 1-Lipschitz on $R^{2}$.

We next establish the relation between $F_{i}$ and the Euclidean distance to the obstacle. For any $q\in O_{i}$, (13) gives

$$\ell_{il}\left(q\right)\leq 0,\;l\in N_{i}.$$

Together with $∥a_{il}∥=1$, this yields

(30) $$\ell_{il}\left(p\right)=\ell_{il}\left(q\right)+a_{il}^{T}\left(p-q\right)\leq a_{il}^{T}\left(p-q\right)\leq ∥p-q∥.$$

Taking the maximum over $l\in N_{i}$ and then the infimum over $q\in O_{i}$ gives

(31) $$\max_{l\in N_{i}}\ell_{il}\left(p\right)\leq \mathrm{dist}\left(p,O_{i}\right).$$

Combining (31) with the upper bound in (26) gives

(32) $$F_{i}\left(p\right)\leq \mathrm{dist}\left(p,O_{i}\right).$$

Choose a center-to-obstacle design clearance $\delta_{b}>\delta$ for the obstacle avoidance reference curve.

Therefore,

(33) $$\left\{p:F_{i}\left(p\right)\geq \delta_{b}\right\}\subseteq \left\{p:\mathrm{dist}\left(p,O_{i}\right)\geq \delta_{b}\right\}\subseteq \left\{p:\mathrm{dist}(p,O_{i})\geq \delta \right\}.$$

Accordingly, a level shift of $F_{i}$ is introduced by defining

(34) $$V_{i}\left(p\right)=F_{i}\left(p\right)-\delta_{b}.$$

Then, (33) can be equivalently written as

(35) $$\left\{p:V_{i}\left(p\right)\geq 0\right\}\subseteq \left\{p:\mathrm{dist}\left(p,O_{i}\right)\geq \delta_{b}\right\}\subseteq \left\{p:\mathrm{dist}(p,O_{i})\geq \delta \right\}.$$

Thus, $V_{i}\left(p\right)\geq 0$ imposes the conservative design clearance $\delta_{b}$ and, consequently, satisfies the physical safety requirement in (16). The zero level set of $V_{i}$ is defined as the obstacle avoidance reference curve

$$\Gamma_{i}=\left\{p:V_{i}\left(p\right)=0\right\}=\left\{p:F_{i}\left(p\right)=\delta_{b}\right\}.$$

Here, $F_{i}$ is the LSE-based obstacle function, and $V_{i}$ is its level-shifted form used to define $\Gamma_{i}$. Neither function is an exact signed-distance function.

To compare the region bounded by the LSE-defined reference curve $\Gamma_{i}$ with the polygonal half-space offset, applying (26) at level $\delta_{b}$ gives

(36) $$\left\{p:m_{i}\left(p\right)\leq \delta_{b}\right\}\subseteq \left\{p:F_{i}\left(p\right)\leq \delta_{b}\right\}\subseteq \left\{p:m_{i}\left(p\right)\leq \delta_{b}+\eta_{i}\right\}.$$

Consequently, every $p\in \Gamma_{i}$ satisfies

$$\delta_{b}\leq m_{i}\left(p\right)\leq \delta_{b}+\eta_{i}.$$

For a fixed polygon and position p, differentiating (23) with respect to $\kappa_{i}$ yields

(37) $$\frac{\partial F_{i}\left(p\right)}{\partial \kappa_{i}}=\frac{1}{\kappa_{i}^{2}}\sum_{l\in N_{i}}\lambda_{il}\left(p\right)\ln\left(N_{i}\lambda_{il}\left(p\right)\right)\geq 0,$$

because the sum is the nonnegative relative entropy of the weights $\lambda_{il}$ with respect to the uniform distribution on the $N_{i}$ edges. Thus, for fixed $\delta_{b}$, increasing $\kappa_{i}$ makes the enclosed region $\left\{p:F_{i}\left(p\right)\leq \delta_{b}\right\}$ shrink monotonically, whereas decreasing $\kappa_{i}$ produces a more conservative enclosure. As $\kappa_{i}\to \infty$, $F_{i}$ converges uniformly to $m_{i}$, and $\Gamma_{i}$ approaches the boundary $m_{i}\left(p\right)=\delta_{b}$ obtained by shifting each polygonal supporting half-space outward by $\delta_{b}$. Given a target function-value approximation tolerance ${\bar{\eta }}_{i}>0$, it suffices to choose

(38) $$\kappa_{i}\geq \frac{\ln N_{i}}{{\bar{\eta }}_{i}}.$$

The physical clearance follows from (35). On $\Gamma_{i}$ and throughout the region $V_{i}\left(p\right)\geq 0$, the robot-center distance satisfies $\mathrm{dist}\left(p,O_{i}\right)\geq \delta_{b}$. For the disk robot of radius $r_{R}$, the distance $d_{\mathrm{edge},i}$ between the robot boundary and the obstacle therefore satisfies

(39) $$d_{\mathrm{edge},i}\left(p\right)=\mathrm{dist}\left(p,O_{i}\right)-r_{R}\geq \delta_{b}-r_{R}=d_{s}+\left(\delta_{b}-\delta \right),\;\delta =r_{R}+d_{s}.$$

Hence, $\delta_{b}$ specifies the guaranteed center-to-obstacle clearance lower bound, while $\delta_{b}-r_{R}$ is the corresponding disk edge-to-edge clearance lower bound. The parameter $\kappa_{i}$ controls the additional conservatism introduced by LSE smoothing relative to the polygonal half-space representation. For every $\kappa_{i}>0$, these lower bounds remain valid whenever $F_{i}\left(p\right)\geq \delta_{b}$ is satisfied. The center-to-obstacle distance along $\Gamma_{i}$ may nevertheless vary with $\kappa_{i}$.

If $p\in \Gamma_{i}$ has its closest obstacle point in the interior of an edge, then $m_{i}\left(p\right)=\mathrm{dist}\left(p,O_{i}\right)$, and

$$\delta_{b}\leq \mathrm{dist}\left(p,O_{i}\right)\leq \delta_{b}+\eta_{i}.$$

Near vertices, $m_{i}\left(p\right)$ and $\mathrm{dist}(p,O_{i})$ generally differ, so (36) alone does not give an upper bound on the center-to-obstacle distance along $\Gamma_{i}$. Even the limiting half-space offset $\left\{p:m_{i}\left(p\right)\leq \delta_{b}\right\}$ can extend beyond the Euclidean offset $\left\{p:\mathrm{dist}\left(p,O_{i}\right)\leq \delta_{b}\right\}$ near vertices. Thus, the limit $\kappa_{i}\to \infty$ eliminates the LSE approximation error but does not generally eliminate this conservatism of the polygonal offset.

**Assumption** **2.** *For every i∈M, there exist constants $\sigma_{i}>0$ and $g_{i}>0$ such that the closed level-set neighborhood of the reference curve $\Gamma_{i}$*

(40) $$B_{i}=\{p:|V_{i}\left(p\right)|\leq \sigma_{i}\}$$

*satisfies*

(41) $$∥\nabla V_{i}\left(p\right)∥\geq g_{i},\;\forall p\in B_{i}.$$

*Here, $\sigma_{i}$ specifies the admissible level-set error defining this neighborhood, and $g_{i}$ is a positive lower bound on the gradient norm within this neighborhood [23,24].*

Since $V_{i}\left(p\right)=F_{i}\left(p\right)-\delta_{b}$, it follows that

$$\nabla V_{i}\left(p\right)=\nabla F_{i}\left(p\right),\;\nabla^{2}V_{i}\left(p\right)=\nabla^{2}F_{i}\left(p\right).$$

The gradient condition in Assumption 2 can be verified constructively for any $0<\sigma_{i}<\delta_{b}$. Fix a point $q_{i}\in \mathrm{int}O_{i}$ for each obstacle *i*, which exists by Assumption 1. All signed half-space values at $q_{i}$ are negative, so the normalized LSE in (23) satisfies $F_{i}\left(q_{i}\right)<0$. Define

(41a) $$C_{i}=\left\{p:\max_{l\in N_{i}}\ell_{il}\left(p\right)\leq \delta_{b}+\sigma_{i}+\eta_{i}\right\},\;D_{i}=\max_{p\in C_{i}}∥p-q_{i}∥.$$

The LSE bound in (26) gives $B_{i}\subseteq C_{i}$. The outward shifts of the half-space constraints leave their recession cone unchanged, so $C_{i}$ is bounded because $O_{i}$ is bounded. Since $O_{i}\subseteq C_{i}$ has a nonempty interior, $0<D_{i}<\infty$, and $D_{i}$ can be computed as the maximum distance from $q_{i}$ to the vertices of $C_{i}$. For every $p\in B_{i}$, convexity gives

$$\nabla F_{i}{\left(p\right)}^{T}\left(p-q_{i}\right)\geq F_{i}\left(p\right)-F_{i}\left(q_{i}\right)\geq \delta_{b}-\sigma_{i}-F_{i}\left(q_{i}\right)>0.$$

By the Cauchy–Schwarz inequality and $∥p-q_{i}∥\leq D_{i}$, it follows that

$$∥\nabla V_{i}\left(p\right)∥=∥\nabla F_{i}\left(p\right)∥\geq \frac{\delta_{b}-\sigma_{i}-F_{i}\left(q_{i}\right)}{D_{i}}>0,\;p\in B_{i}.$$

Therefore, Assumption 2 is satisfied by choosing

(41b) $$g_{i}=\frac{\delta_{b}-\sigma_{i}-F_{i}\left(q_{i}\right)}{D_{i}}.$$

This is a computable, conservative lower bound, rather than necessarily the exact minimum gradient norm on $B_{i}$.

Thus, $B_{i}$ contains no stationary point, and the unit normal and curvature of the reference curve $\Gamma_{i}$ are well defined. For $p\in B_{i}$, define

$$n_{i}\left(p\right)=\frac{\nabla V_{i}\left(p\right)}{∥\nabla V_{i}\left(p\right)∥},\;E_{⊥}=\left[\begin{array}{cc} 0 & -1\\ 1 & 0 \end{array}\right].$$

The vector $n_{i}$ points toward increasing $V_{i}$ and is the outward unit normal to the corresponding sublevel set. The vector $-n_{i}$ points in the opposite direction, toward decreasing $V_{i}$, and is the local inward unit normal. Under the standard Cartesian coordinate convention, $E_{⊥}$ rotates a vector counterclockwise by $90^{{}^{\circ}}$. Thus, for any $p\in B_{i}$, $E_{⊥}n_{i}\left(p\right)$ is tangent to the level set passing through p, $\left\{q\in R^{2}:V_{i}\left(q\right)=V_{i}\left(p\right)\right\}$. In particular, it is a unit tangent to $\Gamma_{i}$ when $p\in \Gamma_{i}$.

Let $K_{i}\left(p\right)$ denote the curvature of $\Gamma_{i}$ at p. The curvature formula for a regular implicit curve gives

(42) $$\left|K_{i}\left(p\right)\right|=\frac{\left|{\left(E_{⊥}n_{i}\left(p\right)\right)}^{T}\nabla^{2}F_{i}\left(p\right)E_{⊥}n_{i}\left(p\right)\right|}{∥\nabla F_{i}\left(p\right)∥}\leq \frac{\kappa_{i}}{g_{i}},\;p\in \Gamma_{i}.$$

The last inequality follows from (28) and Assumption 2.

Define the maximum curvature of the reference curve as

(43) $${\bar{K}}_{i}=\underset{p\in \Gamma_{i}}{\sup}\left|K_{i}\left(p\right)\right|\leq \frac{\kappa_{i}}{g_{i}}.$$

Let $v_{o,i}>0$ denote the obstacle avoidance GVF speed parameter. If the robot were to traverse $\Gamma_{i}$ exactly at the constant speed $v_{o,i}$, the required angular speed would satisfy

$$\left|\omega \right|=v_{o,i}\left|K_{i}\left(p\right)\right|\leq v_{o,i}{\bar{K}}_{i}.$$

Therefore, a sufficient fixed-speed trackability condition is

(44) $$v_{o,i}{\bar{K}}_{i}\leq \min\left\{\omega_{\max},-\omega_{\min}\right\}$$

and (43) yields the more conservative closed-form sufficient condition

(45) $$\frac{v_{o,i}\kappa_{i}}{g_{i}}\leq \min\left\{\omega_{\max},-\omega_{\min}\right\}.$$

The choice of $\kappa_{i}$ therefore involves a tradeoff. A larger $\kappa_{i}$ reduces the conservatism of the LSE representation but may increase the curvature and turning demand near polygon vertices. Since the gradient lower bound $g_{i}$ also depends on $\kappa_{i}$ and the neighborhood $B_{i}$, parameter selection should satisfy the approximation error requirement in (38) together with the turning feasibility condition in (44) or its conservative test in (45).

Condition (45) is an optional offline design test rather than a requirement for the feasibility of the constrained MPC problem. If it is violated, the exact traversal of $\Gamma_{i}$ at the constant speed $v_{o,i}$ is not guaranteed. The bounded-input controller may instead reduce the realized speed or incur a temporary curve tracking error.

### 3.2. Goal and Obstacle Avoidance GVFs

The LSE obstacle model developed above is now used to construct the goal and obstacle avoidance GVFs. The former drives the robot toward the goal when its motion is not restricted by obstacles. The latter combines normal convergence with tangential traversal to maintain a persistent circumnavigation direction near an obstacle.

#### 3.2.1. Goal GVF

Using the goal distance defined in (18), define the goal position error as

(46) $$e_{g}\left(p\right)=p-p_{g}.$$

Choose a goal attraction gain $k_{g}>0$ and a goal GVF speed parameter $v_{g}>0$. For a scalar distance argument r≥0, define the gain function

(47) $$a_{g}\left(r\right)=\left\{\begin{array}{cc} \frac{2}{\pi }\frac{\tan^{-1}\left(k_{g}r\right)}{r}, & r>0,\\ \frac{2k_{g}}{\pi }, & r=0, \end{array}\right.\;k_{g}>0.$$

The definition at r=0 ensures that $a_{g}$ is continuous on r≥0. The goal GVF is defined as

(48) $$\phi_{g}\left(p\right)=-v_{g}a_{g}\;\left(r_{g}\left(p\right)\right)e_{g}\left(p\right),\;v_{g}>0.$$

Its magnitude satisfies

(49) $$∥\phi_{g}\left(p\right)∥=\frac{2v_{g}}{\pi }\tan^{-1}\;\left(k_{g}r_{g}\left(p\right)\right)<v_{g}.$$

Therefore, (49) establishes that $∥\phi_{g}\left(p\right)∥<v_{g}$ for all p∈W.

To characterize the convergence properties of the goal GVF, consider its integral curves generated by

$$\dot{p}=\phi_{g}\left(p\right).$$

For $r_{g}\left(p\left(t\right)\right)>0$, the derivative of the goal distance satisfies

(50) $$\frac{d}{dt}r_{g}\left(p\left(t\right)\right)=\frac{e_{g}^{T}\left(p\left(t\right)\right)}{r_{g}\left(p\left(t\right)\right)}\dot{p}\left(t\right)=-\frac{2v_{g}}{\pi }\tan^{-1}\;\left(k_{g}r_{g}\left(p\left(t\right)\right)\right)<0.$$

**Figure 1:**
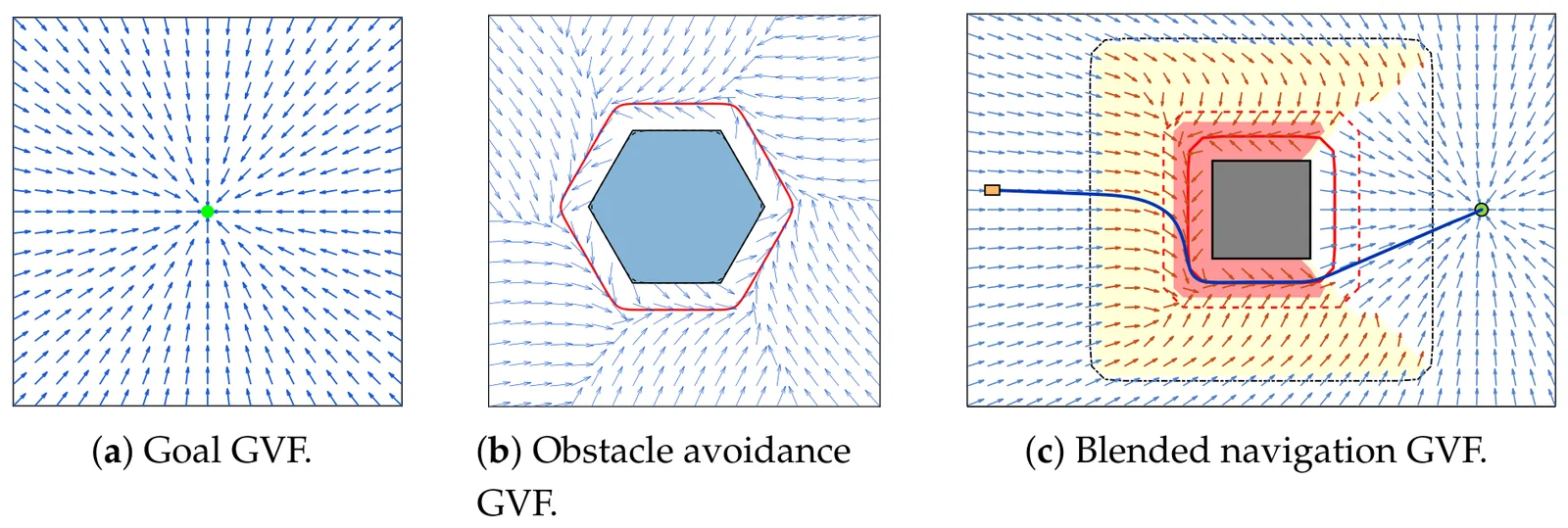
GVF construction. The red curve denotes the LSE obstacle avoidance reference curve $\Gamma_{i}$, and the blue curve denotes a representative integral curve.

Thus, $r_{g}\left(p\left(t\right)\right)$ decreases strictly whenever $p\neq p_{g}$. Moreover, $\phi_{g}\left(p_{g}\right)=0$, and $p_{g}$ is the unique equilibrium of the goal GVF. Consequently, the integral curves generated by the goal GVF converge asymptotically to the goal position $p_{g}$. An example of the goal GVF is shown in Figure 1a.

#### 3.2.2. Obstacle Avoidance GVF

Let $\chi_{i}\in \left\{-1,+1\right\}$ specify the circulation direction around obstacle *i*. With the outward normal and positive $90^{{}^{\circ}}$ rotation defined above, $\chi_{i}=+1$ gives counterclockwise circulation and $\chi_{i}=-1$ gives clockwise circulation. Under Assumption 2, define the oriented unit tangent for $p\in B_{i}$ as

(51) $$t_{i}\left(p\right)=\chi_{i}E_{⊥}n_{i}\left(p\right).$$

For a gain $k_{o,i}>0$ and a scalar level-set error e∈R, define the normal and tangential gain functions

(52a) $$c_{i}\left(e\right)=\frac{2}{\pi }\tan^{-1}\left(k_{o,i}e\right),$$

(52b) $$s_{i}\left(e\right)=\sqrt{1-c_{i}^{2}\left(e\right)},\;k_{o,i}>0.$$

The obstacle avoidance GVF is then constructed as

(53) $$\phi_{o,i}\left(p\right)=v_{o,i}\left[-c_{i}\left(V_{i}\left(p\right)\right)n_{i}\left(p\right)+s_{i}\left(V_{i}\left(p\right)\right)t_{i}\left(p\right)\right].$$

Because

$$n_{i}^{T}\left(p\right)t_{i}\left(p\right)=0,\;c_{i}^{2}\left(V_{i}\left(p\right)\right)+s_{i}^{2}\left(V_{i}\left(p\right)\right)=1,$$

the field has a constant magnitude

(54) $$∥\phi_{o,i}\left(p\right)∥=v_{o,i}.$$

Since $c_{i}\left(e\right)$ is odd and has the same sign as *e*, the normal component of (53) directs the state toward $\Gamma_{i}$ from either side. Indeed, along $\dot{p}=\phi_{o,i}\left(p\right)$,

(55) $${\dot{V}}_{i}\left(p\right)=\nabla V_{i}^{T}\left(p\right)\phi_{o,i}\left(p\right)=-v_{o,i}c_{i}\left(V_{i}\left(p\right)\right)∥\nabla V_{i}\left(p\right)∥.$$

For $V_{i}>0$, the normal component points opposite to the outward unit normal $n_{i}$, so ${\dot{V}}_{i}<0$. For $V_{i}<0$, it points along $n_{i}$, so ${\dot{V}}_{i}>0$. The tangential component is tangent to the local level set and does not contribute to ${\dot{V}}_{i}$.

When $V_{i}\left(p\right)=0$, we have $c_{i}\left(0\right)=0$ and $s_{i}\left(0\right)=1$. Consequently,

(56) $$\phi_{o,i}\left(p\right)=v_{o,i}t_{i}\left(p\right).$$

Therefore, on the reference curve

$$\Gamma_{i}=\left\{p:V_{i}\left(p\right)=0\right\},$$

the obstacle avoidance GVF retains only a nonzero tangential component. Figure 1b shows the resulting obstacle avoidance GVF around $\Gamma_{i}$; the arrows show the direction of the combined field $\phi_{o,i}$ at the sampled positions.

**Proposition** **1.** *Under Assumption 2, with a fixed circulation direction $\chi_{i}$, if the initial position lies within this neighborhood, i.e., $p\left(0\right)\in B_{i}$, then the integral curve generated by*

$$\dot{p}=\phi_{o,i}\left(p\right)$$

*remains within this neighborhood $B_{i}$, and the reference-curve error $|V_{i}\left(p\left(t\right)\right)|$ converges exponentially to zero.*

**Proof.**  By (55), the two boundary components of $B_{i}$ satisfy

$${\left.{\dot{V}}_{i}\right|}_{V_{i}=\sigma_{i}}<0,\;{\left.{\dot{V}}_{i}\right|}_{V_{i}=-\sigma_{i}}>0.$$

Thus, the field points strictly into $B_{i}$ on both boundaries, making this neighborhood forward-invariant. For fixed $\chi_{i}$, the field is smooth on an open neighborhood of the compact regular set $B_{i}$, so its integral curves starting in $B_{i}$ extend to all t≥0.

Define $W_{i}\left(p\right)=\frac{1}{2}V_{i}^{2}\left(p\right)$. Since

$$\nabla V_{i}\left(p\right)=∥\nabla V_{i}\left(p\right)∥n_{i}\left(p\right)$$

and $n_{i}^{T}\left(p\right)t_{i}\left(p\right)=0$, the derivative of $W_{i}$ along the integral curve is

(57) $$\begin{array}{cc} \frac{d}{dt}W_{i}\left(p\left(t\right)\right) & =V_{i}\left(p\left(t\right)\right)\nabla V_{i}^{T}\left(p\left(t\right)\right)\phi_{o,i}\left(p\left(t\right)\right)\\ & =-v_{o,i}V_{i}\left(p\left(t\right)\right)c_{i}\left(V_{i}\left(p\left(t\right)\right)\right)∥\nabla V_{i}\left(p\left(t\right)\right)∥. \end{array}$$

Since $V_{i}c_{i}\left(V_{i}\right)>0$ whenever $V_{i}\neq 0$, this derivative is strictly negative away from $\Gamma_{i}$. Because $e\mapsto \tan^{-1}\left(k_{o,i}e\right)/e$ is monotonically decreasing for e>0, and $c_{i}\left(e\right)$ is odd, it follows that

(58) $$e\;c_{i}\left(e\right)\geq \frac{2}{\pi \sigma_{i}}\tan^{-1}\left(k_{o,i}\sigma_{i}\right)e^{2},\;\left|e\right|\leq \sigma_{i}.$$

Define the resulting exponential convergence rate as

(59) $$\zeta_{i}=\frac{2v_{o,i}g_{i}}{\pi \sigma_{i}}\tan^{-1}\left(k_{o,i}\sigma_{i}\right)>0.$$

Combining this inequality with $∥\nabla V_{i}\left(p\right)∥\geq g_{i}$ for $p\in B_{i}$ gives

(60) $$\frac{d}{dt}W_{i}\left(p\left(t\right)\right)\leq -\zeta_{i}V_{i}^{2}\left(p\left(t\right)\right)=-2\zeta_{i}W_{i}\left(p\left(t\right)\right).$$

Since $B_{i}$ is forward-invariant, this inequality holds for all t≥0. The comparison lemma yields

(61) $$\left|V_{i}\left(p\left(t\right)\right)\right|\leq e^{-\zeta_{i}t}\left|V_{i}\left(p\left(0\right)\right)\right|.$$

Therefore, the reference-curve error converges exponentially to zero. □

**Remark** **1.** *This proposition establishes the exponential convergence of the reference-curve error only for the integral curves of the obstacle avoidance GVF. Due to the robot kinematics and input constraints, this result does not apply directly to the robot’s actual motion trajectories.*

### 3.3. Multi-Obstacle GVF Blending

Define the LSE design-safe position domain

(62) $$D_{b}=\left\{p\in W:F_{i}\left(p\right)\geq \delta_{b},\;\forall i\in M\right\}.$$

The blended navigation GVF is constructed on $D_{b}$ for a goal position $p_{g}\in D_{b}$. By (33), this domain satisfies the physical obstacle clearance requirement. Let $d_{\mathrm{in},i}$ and $d_{\mathrm{out},i}$ denote the inner and outer activation thresholds of $F_{i}$, and let $\varepsilon_{a,i}$ denote the direction threshold used in obstacle weighting. Choose these parameters to satisfy

(63) $$\delta_{b}<d_{\mathrm{in},i}<d_{\mathrm{out},i}<\delta_{b}+\sigma_{i},\;0<\varepsilon_{a,i}<1,\;i\in M.$$

The outer activation region of obstacle *i* is defined as

$$\{p\in D_{b}:F_{i}\left(p\right)<d_{\mathrm{out},i}\}.$$

We assume that the goal lies strictly outside all outer activation regions:

$$F_{i}\left(p_{g}\right)>d_{\mathrm{out},i},\;\forall i\in M.$$

For $p\in D_{b}$, define the set of obstacles whose outer activation regions contain p as

(64) $$A\left(p\right)=\{i\in M:F_{i}\left(p\right)<d_{\mathrm{out},i}\}.$$

For every i∈A(p),

$$0\leq V_{i}\left(p\right)<d_{\mathrm{out},i}-\delta_{b}<\sigma_{i}.$$

Thus, all normals and obstacle avoidance GVFs evaluated in the blend lie within their respective regular bands $B_{i}$. The strict inequality in (63) also places the activation boundary $F_{i}=d_{\mathrm{out},i}$ inside $B_{i}$.

In a multi-obstacle environment, the goal GVF $\phi_{g}$ must be blended with the obstacle avoidance GVFs $\phi_{o,i}$. To this end, the circulation direction associated with each obstacle is first selected according to the relative positions of the robot, goal, and obstacle, and the selected direction is held fixed throughout the corresponding avoidance maneuver.

For $p\in D_{b}\setminus \left\{p_{g}\right\}$, define the unit direction toward the goal. For obstacle *i*, define the local inward unit direction $d_{o,i}$ only when $F_{i}\left(p\right)\leq d_{\mathrm{out},i}$, including the entry boundary. These directions are given by

(65) $$d_{g}\left(p\right)=\frac{p_{g}-p}{∥p_{g}-p∥},\;d_{o,i}\left(p\right)=-n_{i}\left(p\right).$$

The goal direction $d_{g}$ need not coincide with the robot’s actual heading. The stored circulation state $\chi_{i}\in \left\{-1,+1\right\}$ is initialized to a fixed, designer-chosen tie-break sign before obstacle *i* first becomes active. Let $0\leq \varepsilon_{\chi }<1$ be the ambiguity threshold, and let $\chi_{i}^{-}$ and $\chi_{i}^{+}$ denote the stored values immediately before and after an entry update. For planar vectors a and b, det[a,b] denotes the determinant of the matrix with these vectors as columns.

When obstacle *i* enters the active set A(p) in (64), or is already active at initialization, update $\chi_{i}$ using the current position as

(66) $$\chi_{i}^{+}=\left\{\begin{array}{cc} \mathrm{sgn}\;\left(\det[d_{g}\left(p\right),d_{o,i}\left(p\right)]\right),\; & \left|\det[d_{g}\left(p\right),d_{o,i}\left(p\right)]\right|>\varepsilon_{\chi },\\ \chi_{i}^{-},\; & \left|\det[d_{g}\left(p\right),d_{o,i}\left(p\right)]\right|\leq \varepsilon_{\chi } \end{array}\right..$$

The second branch retains the stored sign when the current geometry is ambiguous; on the first update, this is the initialized tie-break sign. In a head-on encounter with $d_{g}=d_{o,i}$, the determinant is zero, so this branch applies.

Following the update, $\chi_{i}=\chi_{i}^{+}$ is held fixed while the robot remains in the region. Evaluating (51) with the post-update value $\chi_{i}^{+}$ and using $d_{o,i}\left(p\right)=-n_{i}\left(p\right)$ gives

$$d_{g}^{T}\left(p\right)t_{i}\left(p\right)=\chi_{i}^{+}\det\left[d_{g}\left(p\right),d_{o,i}\left(p\right)\right].$$

Therefore, at the entry update, whenever $|\det\left[d_{g}\left(p\right),d_{o,i}\left(p\right)\right]|>\varepsilon_{\chi }$, (66) guarantees that

(67) $$d_{g}^{T}\left(p\right)t_{i}\left(p\right)=\left|\det[d_{g}\left(p\right),d_{o,i}\left(p\right)]\right|>0.$$

**Figure 2:**
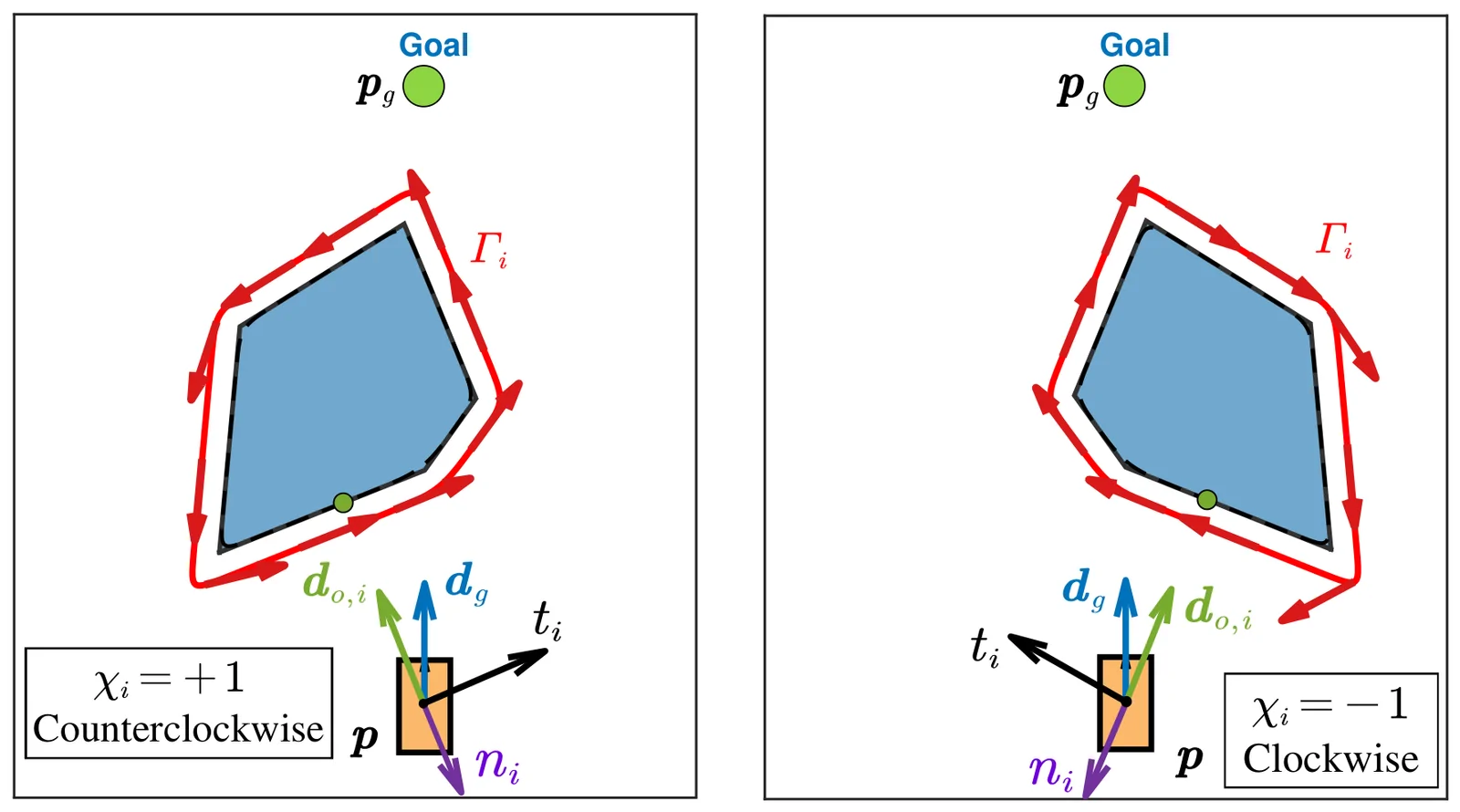
Obstacle bypass direction selection.

Equation (67) certifies a positive goal direction projection only at the selection instant. Figure 2 shows the clockwise and counterclockwise directions represented by $\chi_{i}=-1$ and $\chi_{i}=+1$ in (66).

Holding $\chi_{i}$ fixed preserves the selected clockwise or counterclockwise bypass direction throughout the same avoidance maneuver and prevents repeated reversals caused by local geometric changes. However, because both the goal direction $d_{g}\left(p\right)$ and the local tangent $t_{i}\left(p\right)$ vary as the robot moves, the condition $d_{g}^{T}\left(p\right)t_{i}\left(p\right)>0$ is guaranteed only at the selection instant, not throughout the entire maneuver.

The blended GVF depends on the stored circulation directions as well as the current position. We write it as Φ(p) with these directions held fixed.

To characterize the influence of obstacle *i* at the current position, let S:R→[0,1] be a $C^{\infty }$ nondecreasing cutoff function satisfying

S(z)=0forz≤0,0<S(z)<1for0<z<1,S(z)=1forz≥1.

For $p\in D_{b}\setminus \left\{p_{g}\right\}$ and parameters satisfying (63), define the obstacle weight

(68) $$w_{i}\left(p\right)=\left\{\begin{array}{cc} S\;\left(\frac{d_{\mathrm{out},i}-F_{i}\left(p\right)}{d_{\mathrm{out},i}-d_{\mathrm{in},i}}\right)S\;\left(\frac{\varepsilon_{a,i}-d_{g}^{T}\left(p\right)n_{i}\left(p\right)}{2\varepsilon_{a,i}}\right), & i\in A(p),\\ 0, & i\notin A(p) \end{array}\right..$$

The zero branch is assigned directly without evaluating $n_{i}\left(p\right)$. At $p=p_{g}$, set all obstacle weights to zero and retain the stored circulation directions, without evaluating $d_{g}\left(p\right)$. In (68), the proximity factor is one for $F_{i}\leq d_{\mathrm{in},i}$ and zero for $F_{i}\geq d_{\mathrm{out},i}$, with a smooth transition between them. Hence, $w_{i}=0$ at the outer activation boundary. The direction factor is one when $d_{g}^{T}n_{i}\leq -\varepsilon_{a,i}$ and zero when $d_{g}^{T}n_{i}\geq \varepsilon_{a,i}$, with a smooth transition between. In a goal-directed head-on encounter $d_{g}=-n_{i}$, $w_{i}$ equals the proximity factor and is one if $F_{i}\leq d_{\mathrm{in},i}$. When the goal direction points directly outward, $d_{g}=n_{i}$, the obstacle weight is zero.

For i∈A(p), the robot lies in obstacle *i*’s outer activation region, but $w_{i}\left(p\right)$ may still be zero because of the direction factor. Since both factors can vary along a trajectory, $w_{i}$ need not vary monotonically.

The goal-field weight is

(69) $$w_{0}\left(p\right)=\prod_{i=1}^{M}\left(1-w_{i}\left(p\right)\right).$$

The final navigation GVF is the normalized blend

(70) $$\Phi \left(p\right)=\left\{\begin{array}{cc} \frac{w_{0}\left(p\right)\phi_{g}\left(p\right)+\sum_{i\in A(p)}w_{i}\left(p\right)\phi_{o,i}\left(p\right)}{w_{0}\left(p\right)+\sum_{i=1}^{M}w_{i}\left(p\right)},\; & p\neq p_{g},\\ 0,\; & p=p_{g} \end{array}\right..$$

The sum over an empty A(p) is zero. Obstacles outside this index set are omitted without evaluating $\phi_{o,i}$, including in subsequent weighted sums. For every $p\in D_{b}\setminus \left\{p_{g}\right\}$, because $0\leq w_{i}\left(p\right)\leq 1$, the denominator in the first branch of (70) is strictly positive. If all obstacle weights are zero, then $w_{0}\left(p\right)=1$. Otherwise, at least one obstacle weight is positive. In the former case,

$$\Phi \left(p\right)=\phi_{g}\left(p\right).$$

At $p=p_{g}$, the second branch assigns $\Phi (p_{g})=0$ without evaluating the undefined unit direction $d_{g}\left(p\right)$. Hence, (70) defines $\Phi :D_{b}\to R^{2}$. As the obstacle weights become positive, the corresponding obstacle avoidance GVFs participate gradually through the continuously varying weights, while the contribution of the goal GVF is reduced accordingly. Figure 1c illustrates the resulting blended navigation GVF, showing the transition of the navigation direction between goal approach and obstacle circumnavigation.

For $p\in D_{b}\setminus \left\{p_{g}\right\}$, the first branch of (70) is a normalized convex combination of $\phi_{g}$ and $\phi_{o,i}$ for i∈A(p), with

$$∥\phi_{g}\left(p\right)∥<v_{g},\;∥\phi_{o,i}\left(p\right)∥=v_{o,i},$$

and the second branch is zero. Therefore, the field satisfies

(71) $$∥\Phi \left(p\right)∥\leq \max\left\{v_{g},v_{o,1},\ldots ,v_{o,M}\right\},\;p\in D_{b}.$$

Away from $p_{g}$ and for fixed circulation directions, the LSE functions and their gradients are smooth. At $F_{i}=d_{\mathrm{out},i}$, (63) ensures that the normal and obstacle GVF are smooth in a neighborhood of the boundary, while the $C^{\infty }$ cutoff and all its derivatives vanish on the inactive side. Consequently, each weighted obstacle contribution joins smoothly to its zero extension. Together with Assumption 2 and the strictly positive denominator in (70), this establishes the local Lipschitz continuity of Φ on $D_{b}\setminus \left\{p_{g}\right\}$, relative to this domain. A circulation direction is reselected only after the robot exits the corresponding outer activation region. At such a point, $F_{i}\left(p\right)\geq d_{\mathrm{out},i}$, and the saturation property of *S* gives $w_{i}\left(p\right)=0$. Therefore, a change in $\chi_{i}$ does not introduce a jump in Φ(p).

Since M is finite and each $F_{i}$ is continuous, the strict goal location inequalities ensure that $w_{i}\left(p\right)=0$ for all i∈M and $w_{0}\left(p\right)=1$ in a neighborhood of $p_{g}$. Hence, $\Phi \left(p\right)=\phi_{g}\left(p\right)$ there. The continuity of $\phi_{g}$ and $\phi_{g}\left(p_{g}\right)=0$ imply

$$\lim_{p\to p_{g}}\Phi \left(p\right)=0=\Phi \left(p_{g}\right).$$

Thus, the blended navigation GVF is continuous at the goal.

The nonvanishing of the individual GVFs does not, by itself, guarantee that their blend is nonzero, because fields pointing in opposite directions may cancel exactly. The following proposition provides a regional sufficient condition that excludes such complete cancellation. Let

$$\phi_{0}\left(p\right)=\phi_{g}\left(p\right),\;\phi_{i}\left(p\right)=\phi_{o,i}\left(p\right),\;i\in A\left(p\right).$$

**Proposition** **2.** *Let $R\subset D_{b}\setminus \left\{p_{g}\right\}$ be a region. Suppose that there exists a constant $\eta_{R}>0$ such that, for every p∈R, there exists a unit vector q(p) satisfying*

(72) $$q^{T}\left(p\right)\phi_{i}\left(p\right)\geq \eta_{R},\;\forall i\in \left\{0\right\}\cup A\left(p\right)\;\mathrm{with}\;w_{i}\left(p\right)>0.$$

*Then,*

(73) $$∥\Phi \left(p\right)∥\geq \eta_{R}>0,\;\forall p\in R.$$

*Consequently, the blended GVF cannot vanish through complete cancellation in R.*

**Proof.**  Define the normalized weights

$${\hat{w}}_{i}\left(p\right)=\frac{w_{i}\left(p\right)}{\sum_{j=0}^{M}w_{j}\left(p\right)},\;i=0,\ldots ,M.$$

Then, ${\hat{w}}_{i}\left(p\right)\geq 0$, $\sum_{i=0}^{M}{\hat{w}}_{i}\left(p\right)=1$, and (70) can be written as

$$\Phi \left(p\right)=\sum_{i\in \{0\}\cup A(p)}{\hat{w}}_{i}\left(p\right)\phi_{i}\left(p\right).$$

It follows from (72) that

$$\begin{array}{c} q^{T}\left(p\right)\Phi \left(p\right)=\sum_{i\in \{0\}\cup A(p)}{\hat{w}}_{i}\left(p\right)q^{T}\left(p\right)\phi_{i}\left(p\right)\geq \eta_{R}. \end{array}$$

Since ∥q(p)∥=1, the Cauchy–Schwarz inequality yields

$$∥\Phi \left(p\right)∥\geq q^{T}\left(p\right)\Phi \left(p\right)\geq \eta_{R},$$

which proves (73). □

Equation (66) selects the bypass direction at obstacle entry. In its geometric selection branch, the selected tangent has a positive goal direction projection at that instant. Proposition 2 excludes complete cancellation in a prescribed region when every positively weighted complete GVF has a uniformly positive projection along a common unit direction. This direction may vary with the position and need not coincide with $d_{g}$. The regional condition is independent of the entry selection rule.

**Remark** **2.** *Proposition 2 guarantees nonvanishing only in regions satisfying (72); it does not establish global closed-loop deadlock avoidance or goal convergence.*

## 4. GVF-Based Safe Controller Design

The navigation GVF Φ(p) defined in (70) is used to generate the tracking reference. MPC tracks this reference subject to the robot dynamics, state constraints, and input and input-increment bounds, while DCBF inequalities are imposed as hard constraints in the online optimization. A sufficient condition for closed-loop collision safety is then established by accounting for the one-step DCBF constraint, bounded one-step position prediction error, and inter-sample robot motion.

At sampling instant $t_{k}$, let $x_{j|k}$ and $u_{j|k}$ denote the predicted state and control input at prediction step *j*, respectively, with $x_{0|k}=x_{k}$. Write $x_{j|k}={\left[p_{j|k}^{T},\theta_{j|k}\right]}^{T}$, where $p_{j|k}$ and $\theta_{j|k}$ are the predicted position and heading. The predicted state evolves according to the discrete-time model $f_{d}$ in (6). Let $N\in Z_{\geq 1}$ denote the prediction horizon and let $N_{c}\in Z$, with $1\leq N_{c}\leq N$, denote the control horizon. Only $u_{0|k},\ldots ,u_{N_{c}-1|k}$ are independently optimized. The remaining predicted inputs are defined by

$$u_{j|k}=u_{N_{c}-1|k},\;j=N_{c},\ldots ,N-1.$$

At each sampling instant $t_{k}$, the circulation directions are first updated from the current measured state according to the entry/exit logic in Section 3.3 and then held fixed during the generation of the current reference sequence and MPC optimization. Let $x_{j|k}^{\mathrm{ref}}={\left[{\left(p_{j|k}^{\mathrm{ref}}\right)}^{T},\theta_{j|k}^{\mathrm{ref}}\right]}^{T}$ denote a reference state with position $p_{j|k}^{\mathrm{ref}}$ and heading $\theta_{j|k}^{\mathrm{ref}}$. Set $x_{0|k}^{\mathrm{ref}}=x_{k}$, with $p_{k}\in D_{b}$. The reference positions are generated step by step from the navigation GVF. At each step j=0,…,N−1, generate the candidate reference position as

$${\tilde{p}}_{j+1|k}^{\mathrm{ref}}=p_{j|k}^{\mathrm{ref}}+T_{s}\Phi \left(p_{j|k}^{\mathrm{ref}}\right).$$

Let $\alpha_{j|k}\in \left\{0,1\right\}$ indicate whether this candidate is accepted. If the candidate belongs to $D_{b}$ (i.e., it lies in W and satisfies $F_{i}\left({\tilde{p}}_{j+1|k}^{\mathrm{ref}}\right)\geq \delta_{b}$ for all i∈M), accept it as $p_{j+1|k}^{\mathrm{ref}}$ and set $\alpha_{j|k}=1$. Otherwise, set $p_{\ell |k}^{\mathrm{ref}}=p_{j|k}^{\mathrm{ref}}$ for ℓ=j+1,…,N and $\alpha_{\ell |k}=0$ for ℓ=j,…,N−1. The remaining reference velocities are then zero. Consequently, the resulting virtual reference sequence satisfies

(74) $$p_{j|k}^{\mathrm{ref}}\in D_{b},\;j=0,\ldots ,N.$$

This check ensures only that the virtual reference positions at the prediction instants lie within the domain of the navigation GVF. It does not guarantee the continuous-time collision safety of the robot or the recursive feasibility of the full MPC problem. Robot safety is addressed separately by the subsequent DCBF constraints and Theorem 1 under its stated assumptions.

The reference velocity at prediction step *j* is defined as

(75) $$\nu_{j|k}^{\mathrm{ref}}=\alpha_{j|k}\Phi \left(p_{j|k}^{\mathrm{ref}}\right)=\frac{p_{j+1|k}^{\mathrm{ref}}-p_{j|k}^{\mathrm{ref}}}{T_{s}},\;j=0,\ldots ,N-1,$$

and $\theta_{j+1|k}^{\mathrm{ref}}$ is aligned with $\nu_{j|k}^{\mathrm{ref}}$ when this velocity is nonzero. Otherwise, $\theta_{j+1|k}^{\mathrm{ref}}=\theta_{j|k}^{\mathrm{ref}}$. The resulting state sequence is a nominal tracking reference; the MPC constraints (81d) and (81e) bound the robot’s applied linear and angular speeds and input increments.

Let ${\mathrm{wrap}}_{\left[-\pi ,\pi \right)}\left(\cdot \right)$ map an angle to its unique representative in [−π,π). The corresponding state tracking error is defined as

(76) $$e_{j|k}=\left[\begin{array}{c} p_{j|k}-p_{j|k}^{\mathrm{ref}}\\ {\mathrm{wrap}}_{\left[-\pi ,\pi \right)}\left(\theta_{j|k}-\theta_{j|k}^{\mathrm{ref}}\right) \end{array}\right]\in R^{3}.$$

The control-input increments are defined as

$$\Delta u_{0|k}=u_{0|k}-u_{k-1}$$

and

$$\Delta u_{j|k}=u_{j|k}-u_{j-1|k},\;j=1,\ldots ,N_{c}-1.$$

For a symmetric matrix *P*, the notation P≻0 means that *P* is positive definite. For P⪰0, write ${∥z∥}_{P}^{2}=z^{T}Pz$. Choose state tracking and terminal weights $Q,Q_{f}\in R^{3\times 3}$ with Q≻0 and $Q_{f}≻0$ and an input-increment weight $R\in R^{2\times 2}$ with R⪰0.

To track the local reference state and penalize control-input increments according to *R*, define the finite-horizon cost function

(77) $$J_{k}=\sum_{j=0}^{N-1}∥e_{j|k}{∥}_{Q}^{2}+\sum_{j=0}^{N_{c}-1}∥\Delta u_{j|k}{∥}_{R}^{2}+{∥e_{N|k}∥}_{Q_{f}}^{2}.$$

The weighting matrices *Q*, $Q_{f}$, and *R* are used to penalize the state tracking error over the prediction horizon, the terminal state tracking error, and the control-input increments, respectively. The control-input constraints and DCBF constraints are imposed as hard constraints, while the weighting matrices are used to tune the tracking performance and control-input smoothness [28].

The formulation includes no terminal control-invariant set or dedicated terminal controller; the recursive feasibility of the constrained MPC problem is not established here.

The prediction horizon *N*, control horizon $N_{c}$, and DCBF constraint horizon $N_{b}$ specify the state prediction length, control-input optimization length, and prediction range over which the DCBF constraints are imposed, respectively.

To enforce collision safety, use the LSE function $F_{i}$ in (23) and the shifted function $V_{i}$ in (34) to define the safety function associated with obstacle *i* as

(78) $$h_{i}\left(x\right)=V_{i}\left(p\right)=F_{i}\left(p\right)-\delta_{b}.$$

Then, (35) directly gives

$$\left\{x:h_{i}\left(x\right)\geq 0\right\}\subseteq \left\{x:\mathrm{dist}\left(p,O_{i}\right)\geq \delta_{b}\right\}\subseteq \left\{x:\mathrm{dist}(p,O_{i})\geq \delta \right\}.$$

Hence, $h_{i}\left(x\right)\geq 0$ ensures that the distance from the robot center to obstacle $O_{i}$ is no smaller than the design clearance $\delta_{b}$ and therefore satisfies the edge-to-edge safety requirement in (16).

Choose a DCBF prediction horizon $N_{b}\in Z$ with $1\leq N_{b}\leq N$. For each obstacle i∈M, let $\gamma_{i}\in \left(0,1\right]$ be its DCBF parameter and $\rho_{i}\geq 0$ its robustness margin. For each transition generated by (6), impose the tightened DCBF inequality for $j=0,\ldots ,N_{b}-1$:

(79) $$h_{i}\left(x_{j+1|k}\right)\geq \left(1-\gamma_{i}\right)h_{i}\left(x_{j|k}\right)+\rho_{i}.$$

For an exact prediction model, $\rho_{i}=0$ may be used. When a bounded one-step prediction error is present, $\rho_{i}>0$ provides the corresponding robustness margin. The receding-horizon controller applies only the first optimized input. The j=0 instance of (79) constrains the corresponding one-step prediction, whereas choosing $N_{b}>1$ also constrains later predicted transitions. For compactness, define the barrier residual

(80) $$\xi_{i,j|k}=h_{i}\left(x_{j+1|k}\right)-\left(1-\gamma_{i}\right)h_{i}\left(x_{j|k}\right).$$

Then, (79) is equivalently written as $\xi_{i,j|k}\geq \rho_{i}$.

Let the stacked predicted-state and optimized-input vectors be

$$X_{k}={\left[x_{1|k}^{T},\ldots ,x_{N|k}^{T}\right]}^{T}\in R^{3N},\;U_{k}={\left[u_{0|k}^{T},\ldots ,u_{N_{c}-1|k}^{T}\right]}^{T}\in R^{2N_{c}}.$$

At each control update instant $t_{k}$, the finite-horizon optimization determines $X_{k}$ and $U_{k}$ by minimizing (77). The predicted states must satisfy (6) and remain in X. The first $N_{c}$ independently optimized inputs must satisfy the input and input-increment bounds. For every obstacle i∈M, the first $N_{b}$ predicted transitions must also satisfy the DCBF condition (79). The controller is therefore formulated as

(81a) $$\begin{array}{cc} \min_{X_{k},U_{k}}\;\; & J_{k} \end{array}$$

(81b) $$\begin{array}{cccc} s.t.\;\; & x_{j+1|k}=f_{d}\left(x_{j|k},u_{j|k}\right),\; & \; & j=0,\ldots ,N-1, \end{array}$$

(81c) $$\begin{array}{cccc} \; & x_{j|k}\in X,\; & \; & j=1,\ldots ,N, \end{array}$$

(81d) $$\begin{array}{cccc} \; & u_{\min}\leq u_{j|k}\leq u_{\max},\; & \; & j=0,\ldots ,N_{c}-1, \end{array}$$

(81e) $$\begin{array}{cccc} \; & \Delta u_{\min}\leq \Delta u_{j|k}\leq \Delta u_{\max},\; & \; & j=0,\ldots ,N_{c}-1, \end{array}$$

(81f) $$\begin{array}{cccc} \; & \xi_{i,j|k}\geq \rho_{i},\; & \; & \begin{array}{c} j=0,\ldots ,N_{b}-1,\;i\in M \end{array}. \end{array}$$

Here, (81b) enforces the discrete-time robot dynamics, (81c) constrains the predicted states, and (81d) and (81e) impose the input and input-increment bounds. Constraint (81f) imposes the DCBF condition for every obstacle and, because $N_{b}\geq 1$, always includes the first predicted transition j=0.

The reference sequence satisfying (74) is computed before solving (81) and held fixed during the optimization. The GVF is evaluated at the reference positions as in (75). The predicted robot positions enter the tracking errors and DCBF constraints. When a feasible optimal solution of (81) is obtained within the current control update period, only the first element of the optimal input sequence is applied to the robot:

$$u_{k}=u_{0|k}^{★}=\pi_{\mathrm{MPC}}\left(x_{k},u_{k-1}\right).$$

If no feasible solution is obtained within the current control update period, the controller retains the previously applied input: $u_{k}=u_{k-1}$. At the next control update instant, the latest measured state is used to reconstruct the reference sequence and solve (81) again.

For the control input $u_{k}$ applied to the robot at $t_{k}$, define the corresponding one-step predicted state as

$${\hat{x}}_{k+1}=f_{d}\left(x_{k},u_{k}\right).$$

Let ${\hat{p}}_{k+1}$ denote the position component of ${\hat{x}}_{k+1}$. To account for the mismatch between the actual closed-loop position and this prediction, let ${\bar{\varepsilon }}_{p}\geq 0$ be a known upper bound on the one-step position prediction error, and assume that

(82) $$∥p_{k+1}-{\hat{p}}_{k+1}∥\leq {\bar{\varepsilon }}_{p}.$$

From the linear speed bounds in (7), define

$$\bar{v}=\max\{|v_{\min}|,|v_{\max}|\}.$$

**Theorem** **1.** *Suppose that $h_{i}\left(x_{0}\right)\geq 0$ for all i∈M. At every sampling instant $t_{k}$, assume that the applied input $u_{k}$ satisfies (7), the position prediction error satisfies (82), and the corresponding prediction ${\hat{x}}_{k+1}=f_{d}\left(x_{k},u_{k}\right)$ satisfies*

$$h_{i}\left({\hat{x}}_{k+1}\right)\geq \left(1-\gamma_{i}\right)h_{i}\left(x_{k}\right)+\rho_{i},\;i\in M,\;\gamma_{i}\in \left(0,1\right].$$

*If $\rho_{i}\geq {\bar{\varepsilon }}_{p}$ for all i∈M, then, for every $k\in Z_{\geq 0}$,*

(83) $$h_{i}\left(x_{k}\right)\geq 0,\;\mathrm{dist}\left(p_{k},O_{i}\right)\geq \delta_{b}.$$

*Furthermore, if $\delta_{b}\geq \delta +T_{s}\bar{v}$, then*

$$\mathrm{dist}(p\left(t\right),O_{i})\geq \delta ,\;\forall t\geq 0.$$

**Proof.**  The differential bound (29) shows that $F_{i}$ is globally 1-Lipschitz in p. Hence, $h_{i}\left(x\right)=F_{i}\left(p\right)-\delta_{b}$ is also 1-Lipschitz in p. Using the assumed DCBF inequality for the predicted next state and the error bound in (82), we obtain

(84) $$h_{i}\left(x_{k+1}\right)\geq h_{i}\left({\hat{x}}_{k+1}\right)-∥p_{k+1}-{\hat{p}}_{k+1}∥\geq \left(1-\gamma_{i}\right)h_{i}\left(x_{k}\right)+\rho_{i}-{\bar{\varepsilon }}_{p}\geq \left(1-\gamma_{i}\right)h_{i}\left(x_{k}\right).$$

Since $h_{i}\left(x_{0}\right)\geq 0$ and $\gamma_{i}\in \left(0,1\right]$, induction gives

$$h_{i}\left(x_{k}\right)\geq 0,\;\forall k\in Z_{\geq 0}.$$

Equation (35) then gives

$$\mathrm{dist}\left(p_{k},O_{i}\right)\geq \delta_{b}.$$

For any $t\in [t_{k},t_{k+1})$, the 1-Lipschitz property of the distance-to-set function and $\left|v\left(t\right)\right|\leq \bar{v}$ give

(85) $$\mathrm{dist}\left(p\left(t\right),O_{i}\right)\geq \mathrm{dist}\left(p_{k},O_{i}\right)-∥p(t)-p_{k}∥\geq \delta_{b}-\int_{t_{k}}^{t}\left|v\left(\tau \right)\right|\;d\tau \geq \delta_{b}-T_{s}\bar{v}.$$

Combining (85) with $\delta_{b}\geq \delta +T_{s}\bar{v}$ yields $\mathrm{dist}(p\left(t\right),O_{i})\geq \delta$. □

The one-step DCBF inequality in Theorem 1 corresponds to the j=0 instance of (79). Thus, $N_{b}=1$ includes this constraint for feasible MPC solutions, while $N_{b}>1$ also constrains later predicted transitions.

## 5. Numerical and Hardware Validation

To systematically evaluate the proposed navigation control method, numerical simulations and hardware experiments are conducted. The simulations examine the GVF behavior in representative and dense multi-obstacle environments and compare the proposed method with APF and conventional MPC-DCBF methods, including a paired quantitative comparison over 20 obstacle scenarios. Hardware experiments further assess implementation on a physical mobile robot platform and the operation of the obstacle bypass direction selection mechanism.

### 5.1. Numerical Simulations

The numerical simulations were conducted in MATLAB R2023b on a computer equipped with an Intel Core i7-14700H processor. The controller sampled the robot state with period $T_{s}$ and used the discrete-time model $f_{d}$ in (6) for receding- horizon optimization. In all simulations, each nonconvex obstacle was first decomposed into convex components, which were treated separately in the GVF construction and safety constraints. In the simulations, the robot had a 0.6m×0.4m rectangular footprint. To maintain consistency with the theoretical safety model, we set the robot radius $r_{R}$ to the rectangle’s circumscribed circle radius, rounded upward, so that the disk model encloses the actual robot footprint for every orientation. The principal simulation and control parameters are listed in Table 1. For trajectory results evaluated with the circumscribed disk model, the safety margin remaining after accounting for the robot radius and prescribed buffer is

$$d_{\mathrm{safe}}\left(t\right)=\min_{i\in M}\left[\mathrm{dist}\left(p\left(t\right),O_{i}\right)-r_{R}-d_{s}\right].$$

The geometric safety condition (16) corresponds to $d_{\mathrm{safe}}\geq 0$.

With R=diag(2.0,0), the input increment cost reduces to ${∥\Delta u_{j|k}∥}_{R}^{2}=2{\left(\Delta v_{j|k}\right)}^{2}$. The angular velocity increment is deliberately left unpenalized to preserve steering responsiveness to changes in the GVF reference heading. The angular velocity and its increment remain bounded by the hard constraints in (81d) and (81e).

The parameters in Table 1 do not satisfy the conservative fixed-speed condition (45). Therefore, the simulations evaluate constrained GVF tracking with reference speed adaptation and angular rate saturation, rather than the exact traversal of $\Gamma_{i}$ at the constant speed $v_{o,i}$.

#### 5.1.1. Single-Obstacle and Narrow-Passage Navigation

A diamond-shaped obstacle was first considered to examine the transition between the goal and obstacle avoidance GVFs. The initial state and goal position were

$$x_{0}={\left[-2.5,0,0\right]}^{T},\;p_{g}={\left[2,0\right]}^{T}.$$

**Figure 3:**
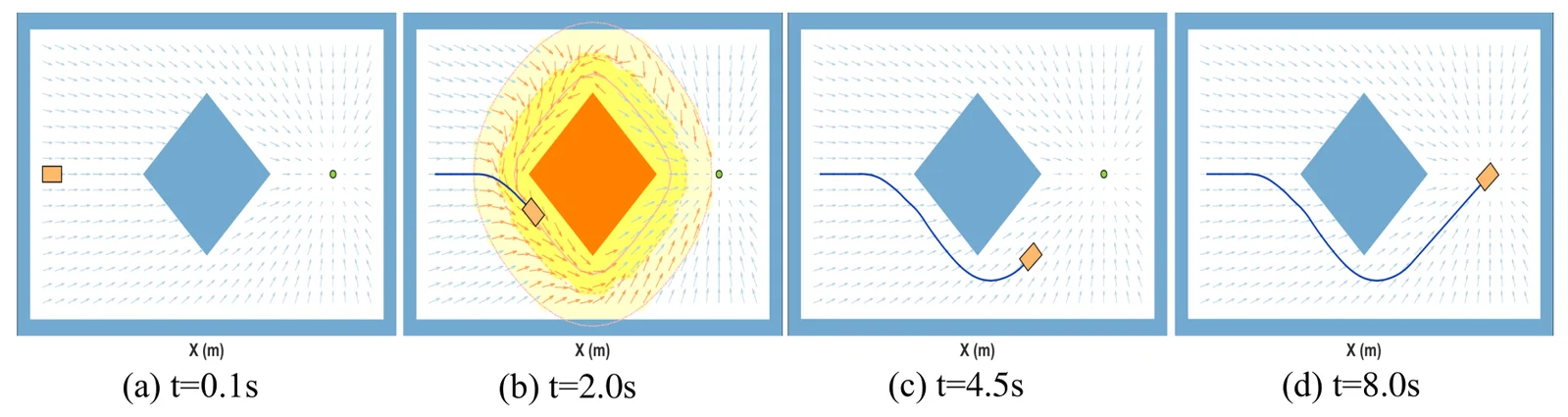
GVF-based navigation in the single-obstacle scenario.

As shown in Figure 3, at t=0.1s, the robot was far from the obstacle, whose influence on the current navigation motion was therefore weak. Consequently, the navigation direction was governed primarily by the goal GVF, and the robot moved toward the goal.

As the robot gradually approached the obstacle blocking its direct motion toward the goal, the influence of the obstacle avoidance GVF increased, and the navigation direction transitioned continuously from direct goal approach to circumnavigation around the obstacle. This transition was evident at approximately t=2.0s. During the circumnavigation phase, the normal component of the obstacle avoidance GVF regulated the robot’s positional deviation from the LSE circumnavigation reference curve, whereas its tangential component sustained motion along the selected circulation direction. Consequently, rather than continuing to move directly toward the goal through the blocking obstacle, the robot maintained a sustained circumnavigation motion around it. After the robot passed the obstacle and gradually left its principal region of influence, the contribution of the obstacle avoidance guiding GVF decreased, and the goal GVF again became dominant. The robot then resumed its motion toward the goal and ultimately entered the goal set $G_{\varepsilon_{g}}$ at approximately t=8.0s. This scenario indicates that the proposed navigation guidance mechanism enables a continuous transition between goal approach and obstacle circumnavigation and restores goal-directed motion after the obstacle has been passed.

**Figure 4:**
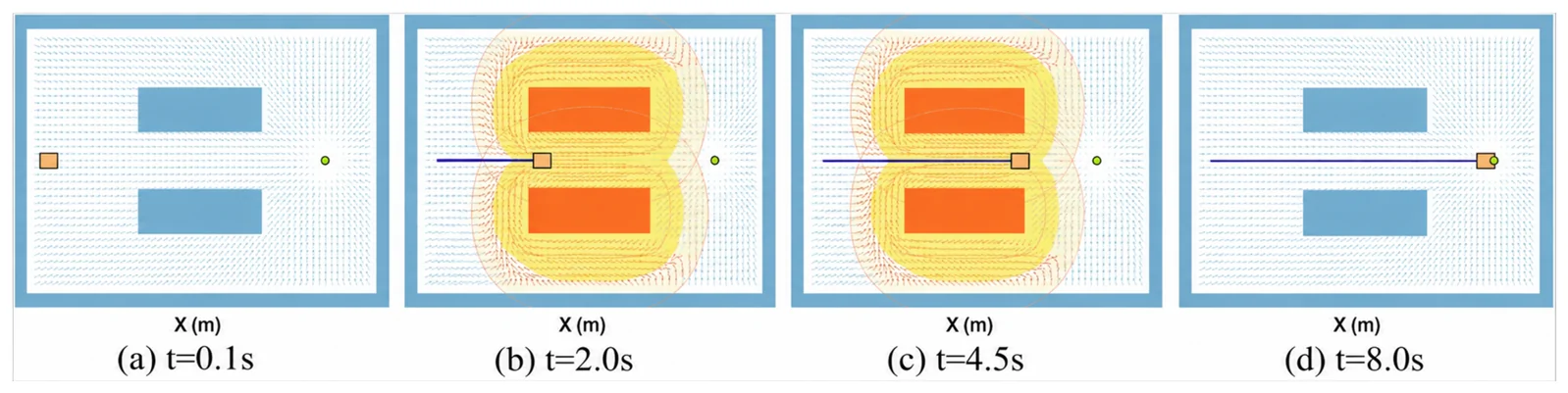
GVF-based navigation in the narrow passage.

The narrow-passage scenario shown in Figure 4 was then considered. Two horizontal rectangular obstacles were located above and below the passage, respectively. The initial state and goal position were set to $x_{0}={\left[-2.5,-0.6,0\right]}^{T},\;p_{g}={\left[2,0.6\right]}^{T}$, respectively. As the robot approached the passage entrance, the upper and lower obstacles simultaneously began to influence the navigation direction and induced opposite circulation directions. Within the passage, the transverse components of the two obstacle avoidance GVFs pointed in opposite directions, whereas their components toward the passage exit were aligned. Consequently, their combined action attenuated the transverse component while preserving an effective forward component along the passage.

This behavior is particularly evident in the field distributions at approximately t=2.0s and t=4.5s. The robot did not stall under the combined influence of the two obstacle avoidance GVFs. Instead, it continued through the narrow passage and entered the goal set at approximately t=8.0s.

#### 5.1.2. Comparative Evaluation Across Obstacle Scenarios

APF, conventional MPC-DCBF, and the proposed method were evaluated in 20 obstacle scenarios, giving 60 algorithm runs. With the workspace and outer boundary fixed, the obstacle counts ranged from one to ten, with two layouts per count and varied obstacle positions and orientations. Each scenario was checked for a traversable route. Scenarios were not selected or removed according to the proposed method’s success. Within each scenario, the methods shared the obstacle map, robot dimensions, initial pose, and goal. Both MPC methods used N=5. All three methods shared the linear and angular velocity bounds listed in Table 1. Both MPC methods used the input-increment bounds |Δv|≤0.45m/s and |Δω|≤0.8rad/s in Table 1. The APF controller applied velocity saturation without additional input-increment constraints.

A run was counted as successful if the robot completed the navigation task without collision. The success rate was the percentage of successful runs among all 20 scenarios. Let *s* denote the trial index in the numerical and hardware evaluations. In this comparison, s∈{1,…,20} identifies the tested scenarios for each method. For a successful run with $K_{s}$ executed control steps, the navigation completion time and path length were

$$T_{\mathrm{nav}}^{\left(s\right)}=K_{s}T_{s},\;L^{\left(s\right)}=\sum_{k=0}^{K_{s}-1}{∥p_{k+1}^{\left(s\right)}-p_{k}^{\left(s\right)}∥}_{2},$$

where $p_{k}^{\left(s\right)}$ is the robot position at step *k*. Here, $T_{\mathrm{nav}}^{\left(s\right)}$ is the elapsed simulation time from the initial state to goal arrival. For the paired comparison, $B^{\left(s\right)}\left(\tau \right)$ is the 0.6m×0.4m rectangular robot footprint at evaluated time τ, and $T_{s}$ includes recorded poses and eight interpolated poses per sampling interval. The minimum robot–obstacle edge-to-edge clearance in scenario *s* is

$$d_{\mathrm{edge},\min}^{\left(s\right)}=\min_{\tau \in T_{s},\;i\in M^{\left(s\right)}}\mathrm{dist}\left(B^{\left(s\right)}\left(\tau \right),O_{i}^{\left(s\right)}\right).$$

As shown in Table 2, the proposed method completed all 20 test tasks, compared with 11 for APF and seven for conventional MPC-DCBF. The path length and navigation completion time were averaged over each method’s successful runs only. Because the sets of successful scenarios differed among the three methods, these metrics describe each method’s path and time characteristics conditional on successful task completion and should be interpreted together with the success rates, rather than being used alone as a direct basis for comparing the overall navigation efficiency across methods.

A separately designed, more complex, dense multi-obstacle environment was then considered to examine successive obstacle-bypassing maneuvers. It contained multiple convex polygonal obstacles and a nonconvex structure composed of several convex polygons, thereby enabling the further evaluation of the proposed method’s ability to perform successive obstacle-bypassing maneuvers in a complex obstacle layout. The initial state and goal position were set to $x_{0}={\left[-8,0,0\right]}^{T},\;p_{g}={\left[7,0\right]}^{T}$, respectively. Under the same initial state, goal position, and obstacle environment, the APF, conventional MPC-DCBF, and proposed methods were evaluated.

**Figure 5:**
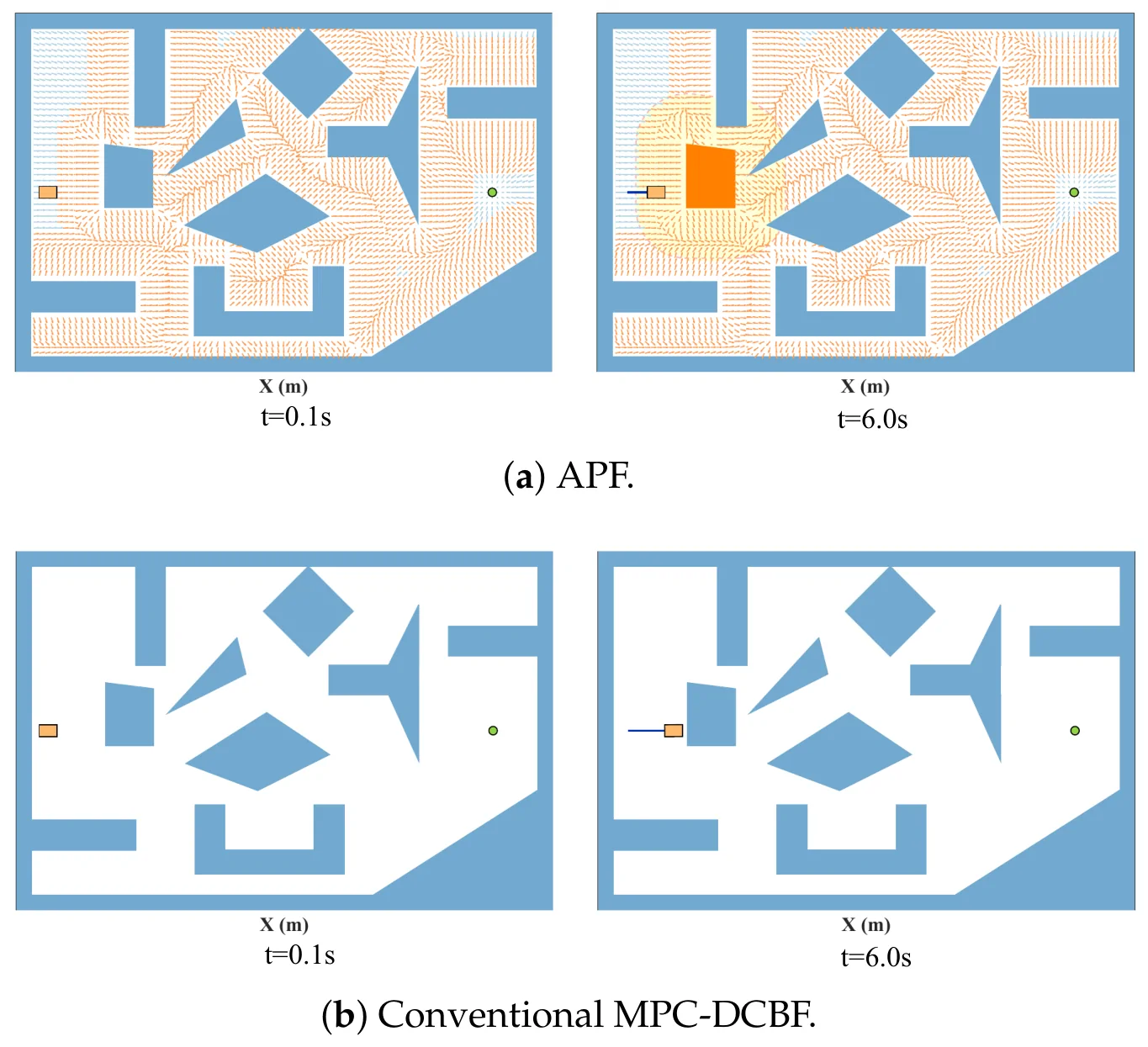
Navigation deadlock of the APF and conventional MPC-DCBF methods in the dense multi-obstacle environment.

For the APF method, as the robot approaches the first blocking obstacle, the goal-attractive and obstacle-repulsive components become nearly antiparallel, causing the magnitude of the resultant guidance vector to decrease substantially. As shown in Figure 5a, at approximately t=6.0s, the robot stops in front of the first blocking obstacle. No collision with the obstacle is observed during this test. Nevertheless, the robot fails to bypass the obstacle and reach the goal, which constitutes a navigation deadlock.

The conventional MPC-DCBF method likewise failed to negotiate the first obstacle blocking the direct path to the goal, as shown in Figure 5b. Under the adopted prediction horizon, control weights, and test conditions, the receding-horizon optimization did not generate a sufficiently persistent tangential maneuver to steer the robot around the obstacle, and the robot eventually came to rest in front of it. Therefore, under this parameter setting, finite-horizon goal-directed optimization combined solely with collision avoidance safety constraints was insufficient to induce an effective obstacle-bypassing maneuver.

To examine whether the navigation failure observed under the baseline N=5 configuration also occurred under several other conventional MPC-DCBF controller configurations, we further tested N=5,10,15,20 in the same dense multi-obstacle environment while retaining $T_{s}=0.05\;s$. In these additional tests, the state tracking weights, terminal weights, input increment penalty, and DCBF-related parameters were also adjusted accordingly. Thus, these tests represent the four controller configurations actually evaluated here, rather than a single-variable comparison in which only the prediction horizon changed. In this dense environment, none of the four tested conventional MPC-DCBF configurations completed the navigation task.

**Figure 6:**
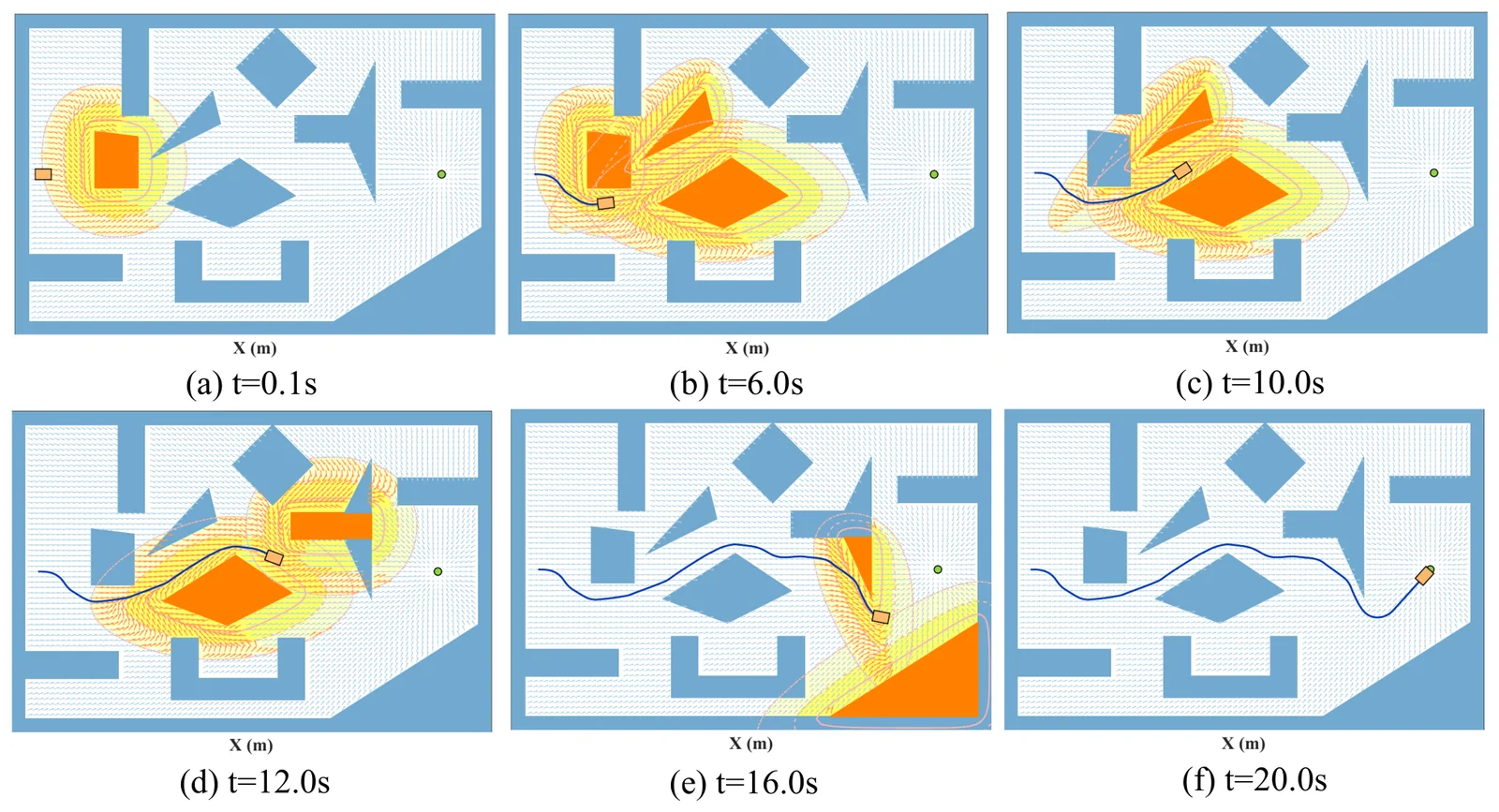
Snapshots of closed-loop navigation using the proposed method in the dense multi-obstacle environment.

Unlike the above two methods, the proposed method explicitly provides an obstacle-bypassing direction through the navigation GVF. When the robot approaches a blocking obstacle, the corresponding obstacle avoidance GVF generates a persistent tangential component, such that the navigation reference is no longer directed solely toward the goal along a direction obstructed by the obstacle. As the robot moves through the environment, different obstacles become active or inactive in the navigation process according to the current local geometry. Consequently, the navigation direction transitions smoothly between goal-directed motion and obstacle-bypassing motion associated with different obstacles. The receding-horizon controller then computes the actual control input according to the current navigation reference and the robot motion constraints, thereby steering the robot along the resulting navigation direction. Figure 6 illustrates the navigation process at six representative time instants. It can be observed that the robot successively bypasses multiple blocking obstacles and eventually enters the neighborhood of the goal. In this dense multi-obstacle test, the robot completes the prescribed navigation task at approximately t=20.0s.

**Figure 7:**
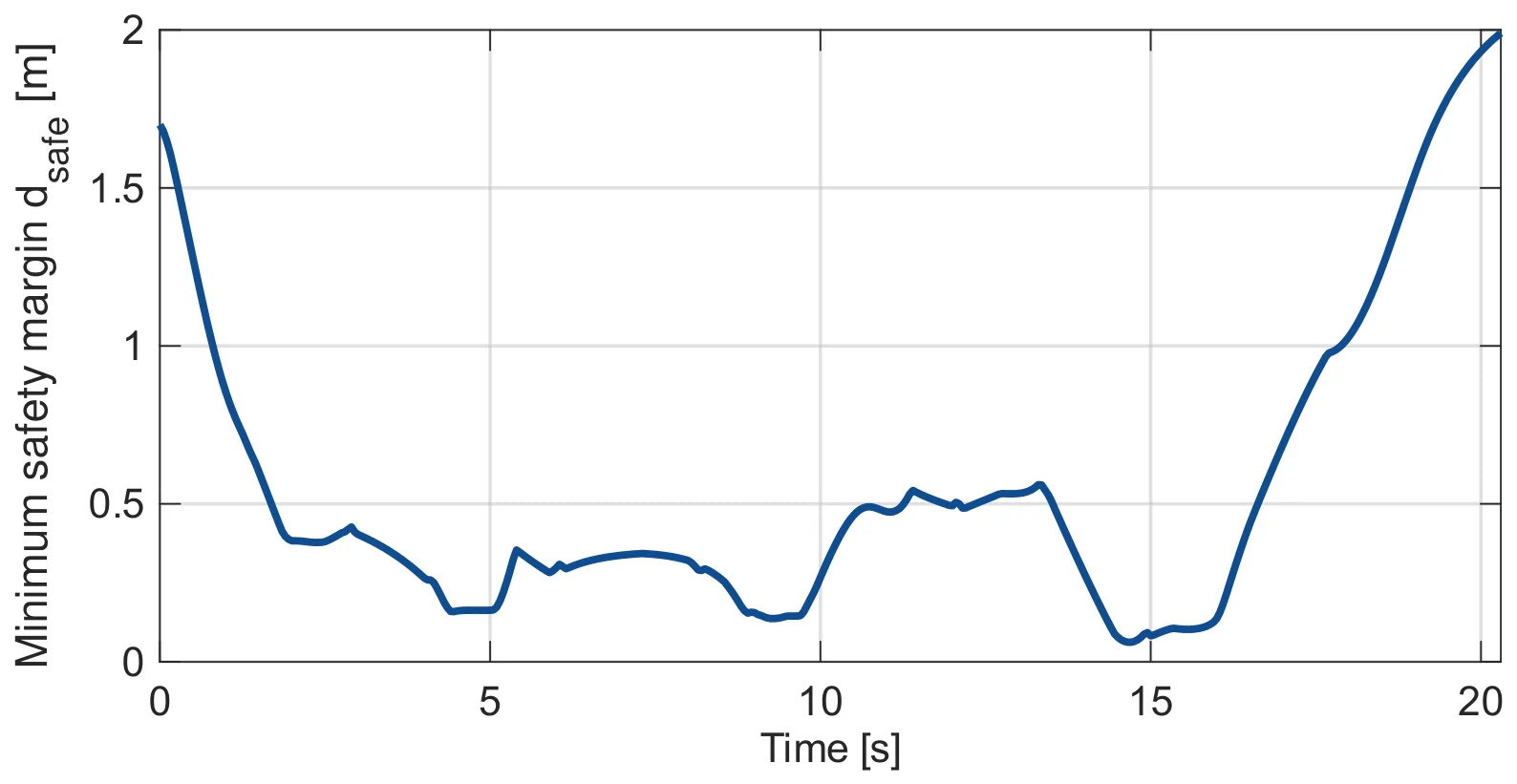
Minimum safety margin $d_{\mathrm{safe}}$ during navigation in the dense multi-obstacle scenario.

Figure 7 shows the evolution of the minimum safety margin $d_{\mathrm{safe}}\left(t\right)$ in the dense multi-obstacle scenario. The safety margin is positive at all recorded instants, with a minimum of 0.0621m, indicating that the robot maintains the prescribed safety buffer $d_{s}=0.1\;m$ and does not collide with any obstacle at these instants.

**Figure 8:**
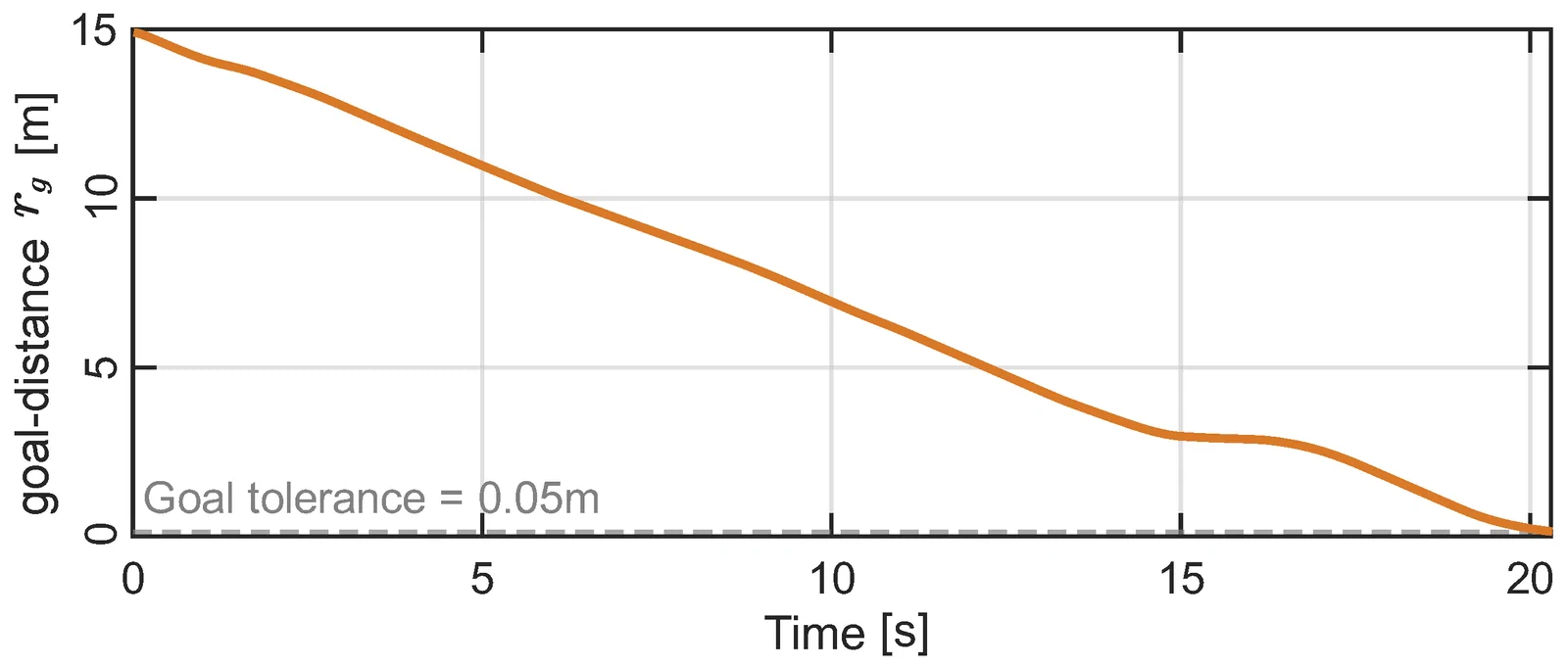
Euclidean distance from the robot to the goal during the dense multi-obstacle simulation.

Figure 8 shows the goal distance $r_{g}\left(p\left(t\right)\right)$ defined in (18). In the complex multi-obstacle environment, the robot-to-goal distance may exhibit local variations during some obstacle avoidance phases. Nevertheless, it shows an overall decreasing trend as the navigation proceeds and eventually satisfies the goal arrival condition adopted for the dense multi-obstacle scenario. Therefore, the robot eventually enters the prescribed goal neighborhood and completes the navigation task.

The outcomes of the three methods in this test are summarized in Table 3.

**Figure 9:**
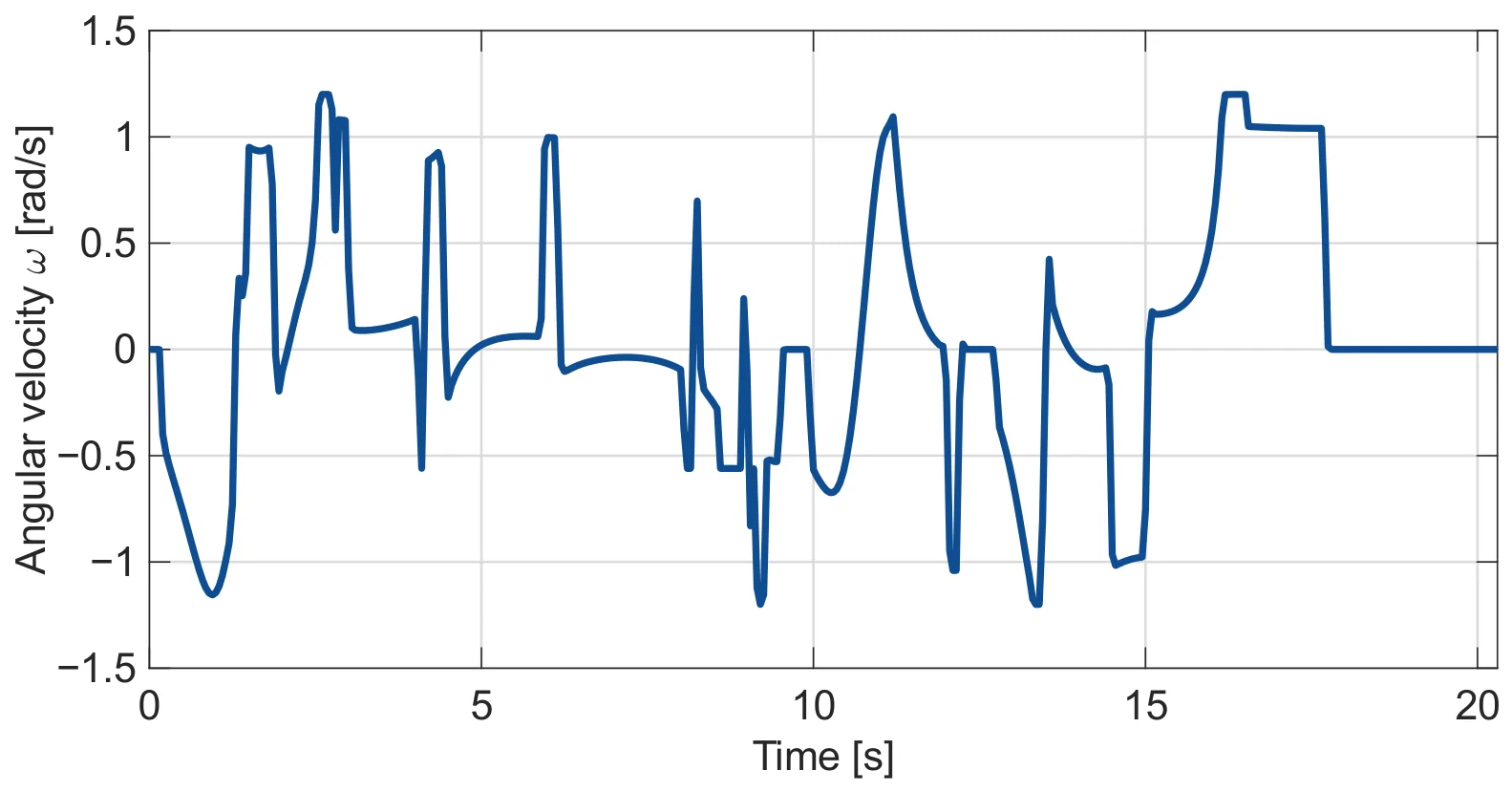
Robot angular velocity during simulated navigation in the dense multi-obstacle scenario.

The angular velocity history in Figure 9 shows rapid steering transients and a limited number of sign reversals during obstacle bypassing, but no sustained high-frequency back-and-forth switching (chattering) was observed in this simulation.

Together with the quantitative comparison in Section 5.1.2, these examples support deadlock-resistant navigation under the tested conditions. The narrow-passage example illustrates how the blended obstacle avoidance directions preserve motion through the passage. In the separately designed dense environment, the proposed method successively bypassed blocking obstacles and reached the goal, whereas APF and the tested conventional MPC-DCBF configurations did not complete the task.

The complete simulation process is available online at https://youtu.be/mNsS6xnZmz4 (accessed on 1 October 2026). The core simulation implementation of the proposed method will be released in the following repository: https://github.com/w694214029/Core-Code-for-the-MDPI-Paper (accessed on 1 October 2026).

### 5.2. Hardware Experiments

#### 5.2.1. Experimental Platform and Onboard Control

Hardware experiments were conducted on a physical mobile robot to evaluate the proposed navigation control method. The Mecanum wheel platform was operated with the lateral velocity command fixed at zero, and the high-level controller generated onlylongitudinal linear velocity and angular velocity commands (v,ω). Previousresearch has analyzed motion errors for the longitudinal motion and in-place rotation of a Mecanum wheel platform [29]; however, these results were obtained on other platforms and are cited here only as background.Under the low-speed experimental conditionsreported here, we used the nonholonomic unicycle model in (2) as an approximate model for high-level prediction and control. A zero lateral velocity command does not imply that the actual lateral velocity is identically zero. In the experiments reported here, we did not directly measure the actual lateral motion, nor did we perform the dedicated quantitative identification of the model mismatch for this platform.

**Figure 10:**
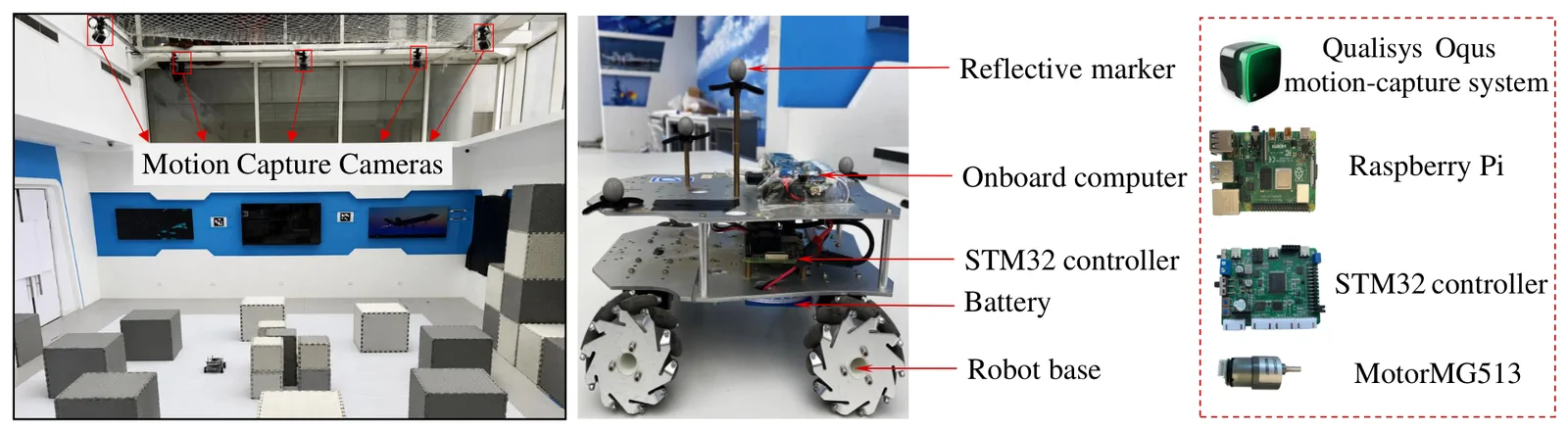
Experimental environment and main hardware components of the physical platform.

The GVF computation, obstacle bypass direction selection, multi-obstacle field blending, and online MPC optimization were executed on an onboard Raspberry Pi running Ubuntu 18.04 and ROS Melodic. The MPC optimization problem in the hardware experiments was solved online on the robot’s onboard Raspberry Pi, using IPOPT as the numerical optimization solver. The Raspberry Pi therefore implemented the complete high-level navigation control pipeline at each control update. Note that the MPC solve times reported below refer only to the time required by IPOPT to solve the MPC optimization problem and do not include other online computation and communication steps, such as GVF updates, reference sequence generation, optimization problem construction, and control command transmission. Robot pose sampling and the MPC prediction model used $T_{s}=0.1\;s$, while the actual control command update period was $T_{\mathrm{ctrl}}=0.5\;s$. If a new admissible command is not available at a scheduled update, the robot retains the previous cycle’s control input until one becomes available. The high-level velocity commands (v,ω) were transmitted to an STM32 Cortex-M4 microcontroller. The STM32 converted these commands into motor commands for the four driven wheels and implemented encoder-feedback PID velocity control to execute the motion commands generated by the high-level navigation controller. An external computer ran QTM, managed the motion capture system, monitored the experiments, and recorded experimental data but did not participate in the GVF computation or online MPC optimization. Hence, the high-level navigation and predictive control computations were performed onboard the robot. The experimental environment and main hardware components of the physical platform are shown in Figure 10.

**Figure 11:**
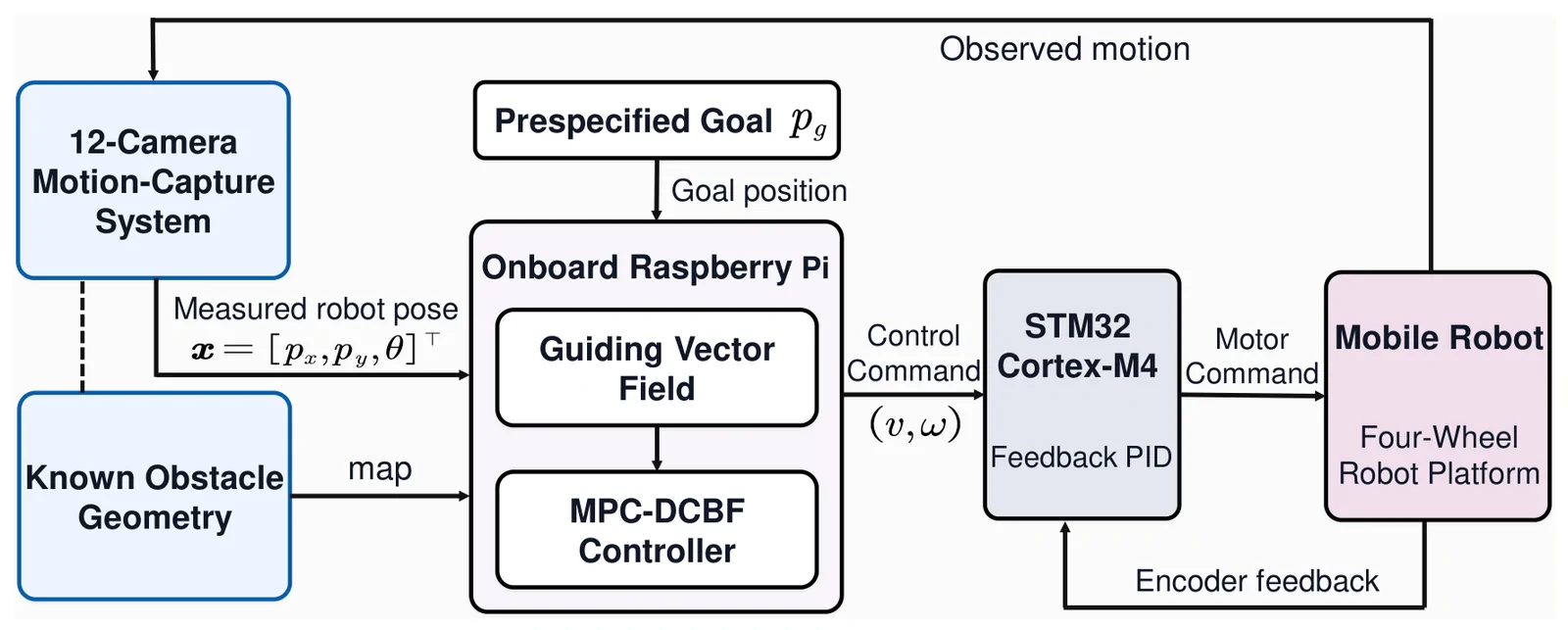
Experimental hardware architecture and closed-loop execution flow.

The hardware configuration and information exchange among the experimental modules are summarized in Figure 11. The goal position and obstacle geometry were specified a priori, the motion capture system provided robot state feedback, and the onboard computing platform and low-level controller performed the high-level navigation control computation and motion command execution, respectively. The initial state and goal position were set to $x_{0}={\left[0,-2.5,\pi /2\right]}^{T},\;p_{g}={\left[0,2.5\right]}^{T}$. Table 4 lists the hardware experiment parameters that differed from the simulation settings.

The one-step position prediction error bound in Table 4 was selected conservatively based on the observed hardware prediction errors. As in the numerical simulations, the hardware parameters do not satisfy the conservative fixed-speed condition (45). Therefore, the exact traversal of $\Gamma_{i}$ at the constant speed $v_{o,i}$ is not claimed.

#### 5.2.2. Optical Motion Capture System

**Figure 12:**
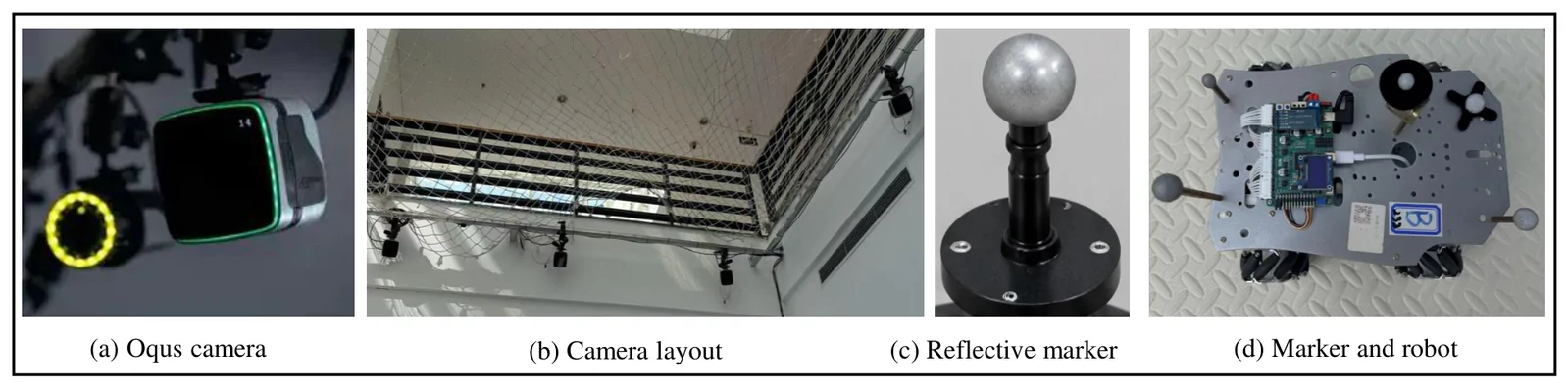
Qualisys Oqus motion capture system and retro-reflective marker configuration for robot state measurement.

Real-time robot state feedback was provided by a 12-camera Qualisys Oqus 3+ optical motion capture system (Qualisys AB, Gothenburg, Sweden). The cameras were arranged around the experimental workspace to provide multi-view coverage and reduce marker occlusion during robot motion. As shown in Figure 12, five retro-reflective markers were rigidly mounted on the upper plate of the robot in an asymmetric configuration. Multiple cameras synchronously observed the markers, and QTM reconstructed their three-dimensional positions from the calibrated multi-camera measurements and obtained the rigid-body position and orientation of the robot. The principal specifications of the motion capture system and markers are summarized in Table 5.

Because the motion capture system operated at 120Hz while robot pose sampling was performed at 10Hz, an updated robot pose measurement was available at every high-level control update. Although QTM provides the complete 6DOF pose of the tracked rigid body, the planar navigation controller uses the robot position $\left(p_{x},p_{y}\right)$ and heading angle θ in the global coordinate frame. At each high-level control update, the latest motion capture pose updated the navigation GVF and served as the current state for MPC. The obstacle geometry and the goal position were specified a priori in the same global frame.

#### 5.2.3. Experimental Results

**Figure 13:**
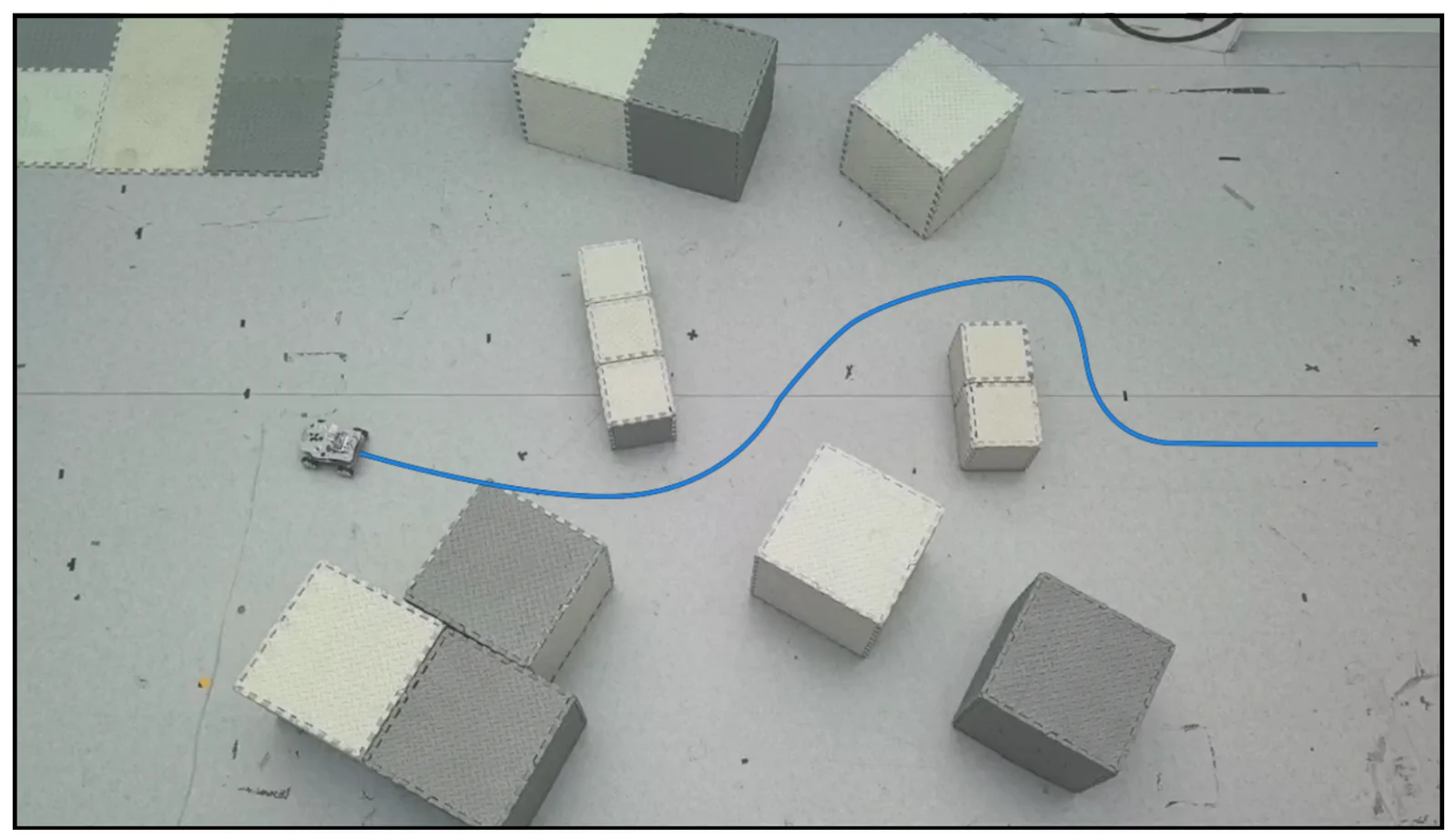
Hardware navigation result of the proposed method in Run 3. The blue curve denotes the robot trajectory.

To assess the repeatability of the hardware results, three navigation experiments were conducted using the same obstacle map, controller parameters, initial state, and goal position. Figure 13 shows the representative trajectory from Run 3 of the proposed method in the multi-obstacle environment. The robot moved from the initial position toward the goal region. When an obstacle was encountered, the bypass direction was determined according to the proposed direction selection rule, and the robot moved around the obstacle along the selected direction. After passing the obstacle, the robot resumed motion toward the goal. As navigation proceeded, the robot successively bypassed multiple obstacles and ultimately reached the goal region.

This study considers point-to-point navigation, with the local reference sequence generated online by the navigation GVF. Navigation accuracy is therefore evaluated using the final goal error rather than the tracking error relative to a predefined reference trajectory. The state tracking error in Section 4 is used within the MPC optimization to penalize deviations from the local reference. The final goal error in each hardware run is defined as

(86) $$e_{g}^{\left(s\right)}={∥p_{\mathrm{final}}^{\left(s\right)}-p_{g}∥}_{2},$$

where $p_{\mathrm{final}}^{\left(s\right)}$ denotes the robot position at the end of that run. Analogously to the edge-to-edge clearance reported in Section 5.1.2, the minimum clearance of the disk model robot from the obstacles over the recorded positions in Run *s* is

(87) $$d_{\mathrm{edge},\min}^{\left(s\right)}=\min_{k}\min_{i\in M}\left[\mathrm{dist}\;\left(p_{k}^{\left(s\right)},O_{i}\right)-r_{R}\right],$$

where $p_{k}^{\left(s\right)}$ is the recorded robot position at sample *k*. This disk model edge-to-edge clearance does not subtract the safety buffer $d_{s}$. The final goal errors, minimum clearances, and onboard MPC solve times for all three runs are summarized in Table 6.

In Run 3, shown in Figure 13, the final goal error was 0.18m, and the robot ultimately satisfied $r_{g}\left(p\right)\leq \varepsilon_{g}=0.2\;m$, entering the prescribed goal region. A run was counted as successful if the robot entered the prescribed goal region without collision. All three runs were successful, giving an experimental success rate of 100% (3/3). All three minimum clearances exceeded $d_{s}+0.05=0.19\;m$, indicating that the prescribed obstacle safety buffer was maintained with more than 0.05m remaining at all recorded positions.

As shown in Table 6, the arithmetic mean of the three run-level mean MPC solve times was 0.385s, whereas the maximum observed solve time was 0.498s. Relative to the 0.5s control command update period, this mean corresponds to a nominal time window of approximately 0.115s for other online operations. The maximum observed solve time should therefore be distinguished from the average computational burden. If a new admissible control input is unavailable at a scheduled update, the robot continues to apply the input from the previous cycle until a new input becomes available, thereby maintaining control-input application during delayed updates.

These timing results were obtained in the complex multi-obstacle environment used in the hardware experiments. When fewer obstacle constraints need to be considered, simpler environments may reduce the computational burden of optimization; however, the solve time also depends on numerical convergence, and such reductions have not been quantified in this study. The observed closed-loop operation, mean solve times, and input retention mechanism together support the practical feasibility of online implementation under the tested conditions. Since the reported times cover only IPOPT optimization, and the end-to-end execution time of the complete control cycle was not measured separately, these results alone do not establish that the method satisfies strict real-time requirements across all operating environments.

The complete hardware navigation experiment for Run 3 is available online at https://youtu.be/sEjDvcsK0u4 (accessed on 1 October 2026). The experimental results show that, with real-time robot pose feedback, the proposed navigation control method can be implemented on a physical mobile robot platform for multi-obstacle navigation and can complete both obstacle bypassing and goal reaching under the reported experimental conditions.

## 6. Conclusions

This paper addresses safe mobile robot navigation in environments with convex polygonal obstacles and proposes a deadlock-resistant navigation framework based on GVFs, MPC, and DCBFs. By constructing and blending the goal GVF and obstacle avoidance GVFs, a regional sufficient condition is established to exclude the complete cancellation of the blended vector field. In addition, a sufficient condition for collision safety is derived by accounting for one-step position prediction errors and inter-sample robot motion. Numerical simulations show that, in the test scenarios wherethe tested APF and conventional MPC-DCBF configurations fail to reach the goal, the proposed method successfully completes the corresponding navigation tasks. Hardware experiments further demonstrate the practical implementability of the proposed method under the reported experimental conditions.

## Figures and Tables

**Table 1:** Simulation and controller parameters.

Parameter	Symbol	Value
Sampling period	$T_{s}$	0.05s
Maximum simulation time	$T_{\max}$	45s
Goal position tolerance	$\varepsilon_{g}$	0.05m
Circumscribed disk radius	$r_{R}$	0.361m
Safety buffer	$d_{s}$	0.1m
Design clearance	$\delta_{b}$	0.52m
Linear speed range	$\left[v_{\min},v_{\max}\right]$	[0,0.9]m/s
Angular speed range	$\left[\omega_{\min},\omega_{\max}\right]$	[−1.2,1.2]rad/s
Maximum linear speed increment	$\Delta v_{\max}$	0.45m/s
Maximum angular speed increment	$\Delta \omega_{\max}$	0.8rad/s
Previously applied input at k=0	$u_{-1}$	${\left[0,0\right]}^{T}$
Goal GVF gain	$k_{g}$	$1.0\;m^{-1}$
Goal GVF speed parameter	$v_{g}$	1.0m/s
Obstacle avoidance GVF gain	$k_{o,i}$	$2.0\;m^{-1}$
Obstacle avoidance GVF speed parameter	$v_{o,i}$	1.0m/s
LSE smoothing parameter	$\kappa_{i}$	$4.0\;m^{-1}$
Inner activation threshold	$d_{\mathrm{in},i}$	0.70m
Outer activation threshold	$d_{\mathrm{out},i}$	1.00m
Bypass direction selection threshold	$\varepsilon_{\chi }$	0.10
Obstacle activation direction threshold	$\varepsilon_{a,i}$	0.10
Prediction horizon	*N*	5
Control horizon	$N_{c}$	4
DCBF prediction horizon	$N_{b}$	5
DCBF parameter	$\gamma_{i}$	0.20
DCBF robust margin	$\rho_{i}$	0m
One-step position prediction error bound	${\bar{\varepsilon }}_{p}$	0m
State tracking weight	*Q*	diag(20,20,100)
Terminal state weight	$Q_{f}$	diag(30,30,60)
Input increment weight	*R*	diag(2.0,0)

**Table 2:** Quantitative comparison over 20 obstacle scenarios.

Metric	APF	Conventional MPC-DCBF	Proposed Method
Success rate	55% (11/20)	35% (7/20)	100% (20/20)
Minimum edge-to-edge clearance across all scenarios (m)	0.796	0.180	0.161
Mean path length over successful runs (m)	15.773	14.783	14.999
Mean navigation completion time over successful runs (s)	25.909	16.450	17.298
Mean per-step MATLAB computation time (ms)	0.145	90.17	182.384

**Table 3:** Results in the dense multi-obstacle environment.

Method	Collision	Goal	Outcome
APF	No	No	Navigation deadlock at the first blocking obstacle
MPC-DCBF	No	No	Navigation deadlock at the first blocking obstacle
Proposed method	No	Yes	Bypassed multiple obstacles and reached the goal at 20 s

**Table 4:** Hardware experiment parameters.

Parameter	Symbol	Value
Experimental area	W	[−2,2]×[−2.5,2.5]m
Pose sampling/MPC prediction step	$T_{s}$	0.1s
Control command update period	$T_{\mathrm{ctrl}}$	0.5s
Goal position tolerance	$\varepsilon_{g}$	0.2m
Prediction horizon	*N*	8
Maximum linear speed	$v_{\max}$	0.4m/s
Angular speed range	$\left[\omega_{\min},\omega_{\max}\right]$	[−0.8,0.8]rad/s
Maximum linear speed increment	$\Delta v_{\max}$	0.02m/s
Maximum angular speed increment	$\Delta \omega_{\max}$	0.04rad/s
Safety buffer	$d_{s}$	0.14m
Center-distance safety threshold	δ	0.501m
Design clearance	$\delta_{b}$	0.55m
One-step position prediction error bound	${\bar{\varepsilon }}_{p}$	0.1m
DCBF robustness margin	$\rho_{i}$	0.1m

**Table 5:** Motion capture system and marker configuration.

Item	Specification
Sensor resolution	1.3 megapixel (1296×1024)
Maximum full-resolution capture rate	500Hz
Operating capture rate	120Hz
Root mean square position accuracy	<0.01mm
Infrared strobe	850nm
Marker diameter	10mm
Marker material	Retro-reflective

**Table 6:** Quantitative results of three repeated hardware navigation experiments.

Run	Final GoalError (m)	Minimum Clearance(Disk Model, m)	Mean MPCSolve Time (s)	Maximum MPCSolve Time (s)
Run 1	0.19	0.23	0.368	0.498
Run 2	0.16	0.25	0.389	0.485
Run 3	0.18	0.24	0.398	0.490

## Data Availability

The original contributions presented in this study are included in the article. Further inquiries can be directed to the authors.
